# A methodological tool for sustainability and feasibility assessment of indoor vertical farming with artificial lighting in Africa

**DOI:** 10.1038/s41598-023-29027-8

**Published:** 2023-02-06

**Authors:** Ivan Paucek, Emanuele Durante, Giuseppina Pennisi, Stefania Quaini, Giorgio Gianquinto, Francesco Orsini

**Affiliations:** 1 DISTAL – Department of Agricultural and Food Sciences, Alma Mater Studiorum – University of Bologna, Bologna, Italy; 2 FEEM – Foundation Eni Enrico Mattei, Milan, Italy

**Keywords:** Environmental impact, Sustainability

## Abstract

African agriculture is bound to face challenges for its future food systems development and economic transformation. Indoor vertical farms with artificial lighting represent an opportunity that has been gaining relevance worldwide, thanks to their potential to enable high productivity rates, food quality and safety, year-round production, and more sustainable use of water and mineral nutrients. The present study assesses the potential for vertical farming technology integration within the African continent, targeting the countries where a more sustainable approach could be achieved. A deep analysis of each territory’s major opportunities and challenges was built through an updated database of 147 development indicators from 54 African states. Countries such as South Africa, Seychelles, Egypt, Mauritius, Morocco, Tunisia, Algeria, Cape Verde, and Nigeria showed the best prospective for indoor vertical farming implementation. Moreover, Seychelles, South Africa, and Egypt resulted to be the countries where vertical indoor farming could be more sustainable.

## Introduction

The African continent will face considerable challenges for its agriculture development and food systems. While the global population is expected to grow to around 9.7 billion in 2050, only countries of sub-Saharan Africa could account for more than half of this worldwide growth between 2019 and 2050^1^. Besides, it is expected that Africa will have more people living in cities by 2050 than Europe, Latin America, or North America^2^. With the import-dependent staple food market, this trend presents a favorable environment for fostering local food production^3^. Food demand will significantly increase, leading to changes in its supply and security to struggle against undernutrition and to provide a productive and healthy life for the African population^4,5^. Additionally, climate change threatens the African agriculture sector and its water availability^6^. Future projections for Southern Africa indicate reduced rainfall, increased temperatures, and high variability on weather change expecting a scenario of reductions between 15 and 50% in agricultural productivity^6^. Similar estimations are expected for the North African region, where a 1% increase in temperature in winter is expected to result in a 1.12% decrease in agricultural productivity in the area^7^, with the resulting social implications^8^.

Agriculture has been the mainstay of African economies for decades, and today the sector accounts for 61% of employment and represents 25% of African Gross Domestic Product (GDP)^9^. Improved agricultural productivity is a requirement that can support human capital development allowing investments for better nutrition, health, and education^10^ that can directly impact a rapid poverty reduction and stimulate economic incomes/growth^11^. Therefore, it is necessary to transform the agricultural sector in Africa, and a need for investments in the modernization of agriculture is evident. Innovative agriculture technologies can support and accelerate this transformation across the continent^12^ and fight against the current challenges of African agriculture^13^. In this sense, a new way of farming is being developed worldwide to tackle these mentioned trends and to improve future food production sustainably, exemplified as indoor vertical farming with artificial lighting^14^.

Indoor vertical farming is an emerging industry of intensive plant production systems with vertically stacked shelves in a controlled environment with artificial lighting and soilless cultivation systems (e.g., hydroponic, aeroponic, aquaponic)^15^. Food security, high yields, no or reduced pesticides or herbicides requirements, low transportation costs, year-round production, water use efficiency, and resilience to climate change are some of the principal benefits of indoor vertical farms^16^. Despite the confirmed potential of vertical farming, some of its drawbacks preclude its viable application everywhere. It requires for greater investments (infrastructure costs, operational costs, employment of several technologies), proper availability of resources such as energy and water, expertise labor, research action, among other demands to make it feasible^17^. Therefore, a methodology that quantifies the opportunities and challenges of vertical farming in a definite location is needed to evaluate its feasibility in the area, while also focusing on providing a perspective of urban food production addressing sustainability issues. The potentiality of being highly feasible for indoor vertical farming implementation will mean that resources to develop outdoors simplified vertical farming systems with locally available materials and fewer investments will also be possible^18^. Although these simplified systems are not considered in the frame of this research work, it is acknowledged that they also would represent a great potential to develop new forms of cultivation in the less feasible countries.

The present study aims to assess the position of 54 individual African countries concerning their feasibility for implementing the indoor vertical farming industry and their sustainability potential in its application. The feasibility was analyzed by the elaboration of 14 macro-categories organized in 3 macro-areas which grouped 147 African Development Indicators. These indicators were selected from reliable public statistics on the last five years of data available, covering the period 2006–2020. The matrix based on the macro-categories created for the feasibility assessment was adapted to the main dimensions of the vertical farming sector for sustainability interpretation. Consequently, the sustainable assessment can be defined as a statistical measure informing about the viability for vertical farming implementation across the African countries where it is most feasible. Furthermore, four more thematic areas of feasibility analysis (*Urban development*, *Energy*, *Food Security* and *Science and Technology*) were added to the three main pillars of sustainability (Economic, Environmental and Social), according to their importance in vertical farming sustainability. The synthetic development measure (SMR) was the research method used to assess the implementation of vertical farming systems in African countries. The data for calculations were retrieved from World Bank and FAO sources.

## Results

### Overview of macro-categories analyzed

**Figure 1:**
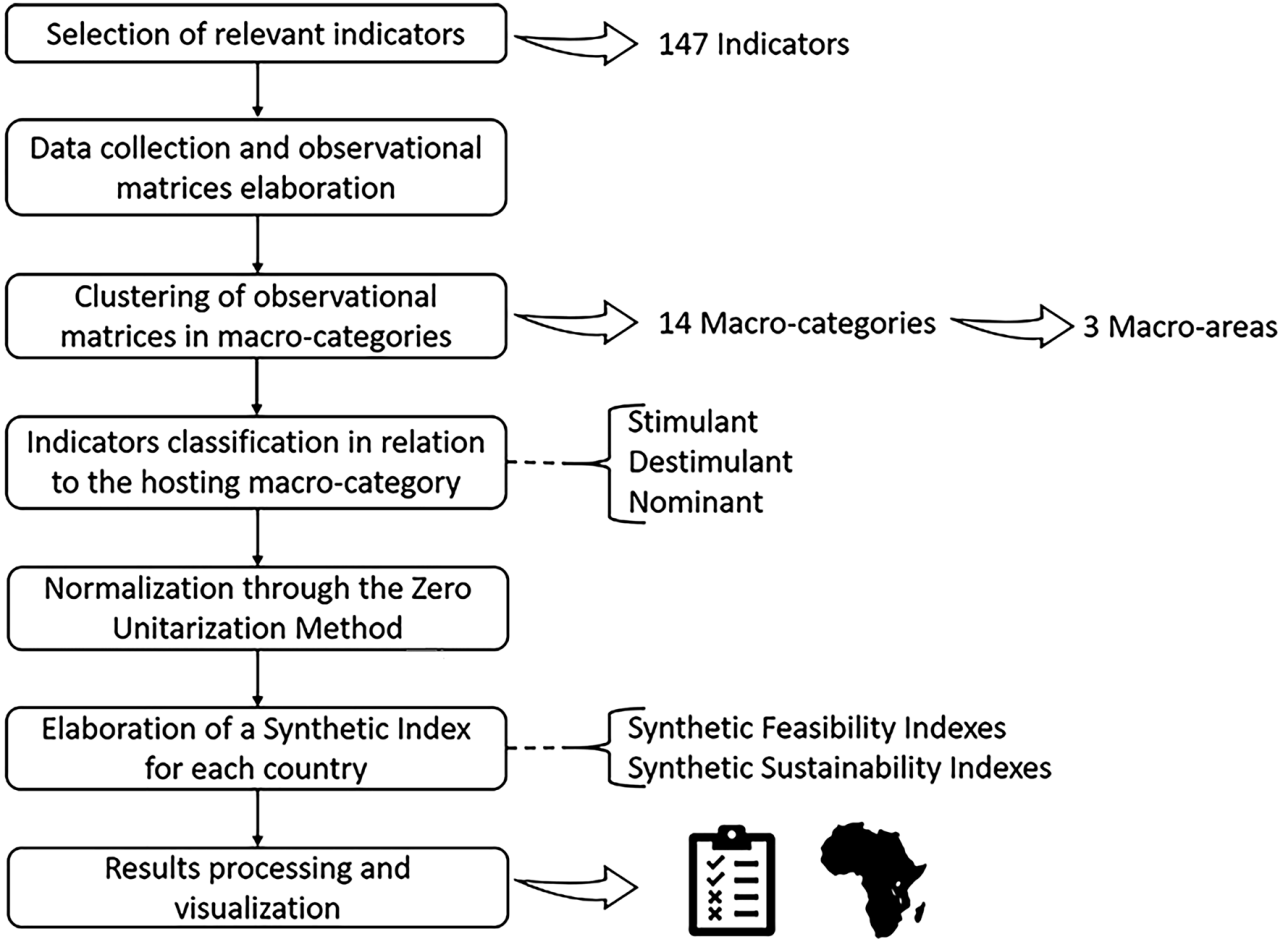
Methodological framework for the feasibility and sustainability assessment of vertical farming in Africa.

**Figure 2:**
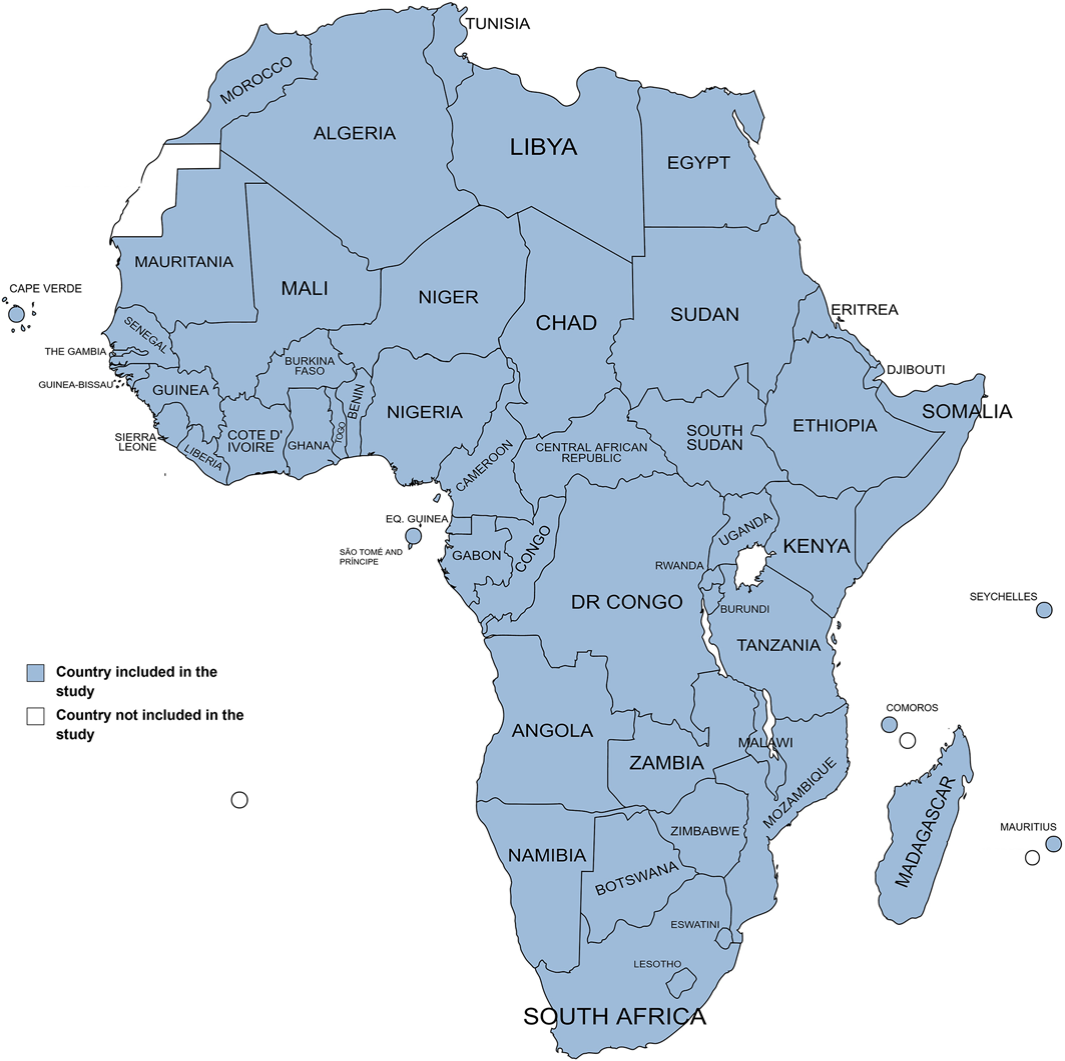
Map illustrating 54 countries selected for feasibility and sustainability assessment of vertical farming in Africa (generated with MapChart, https://www.mapchart.net/).

**Figure 3:**
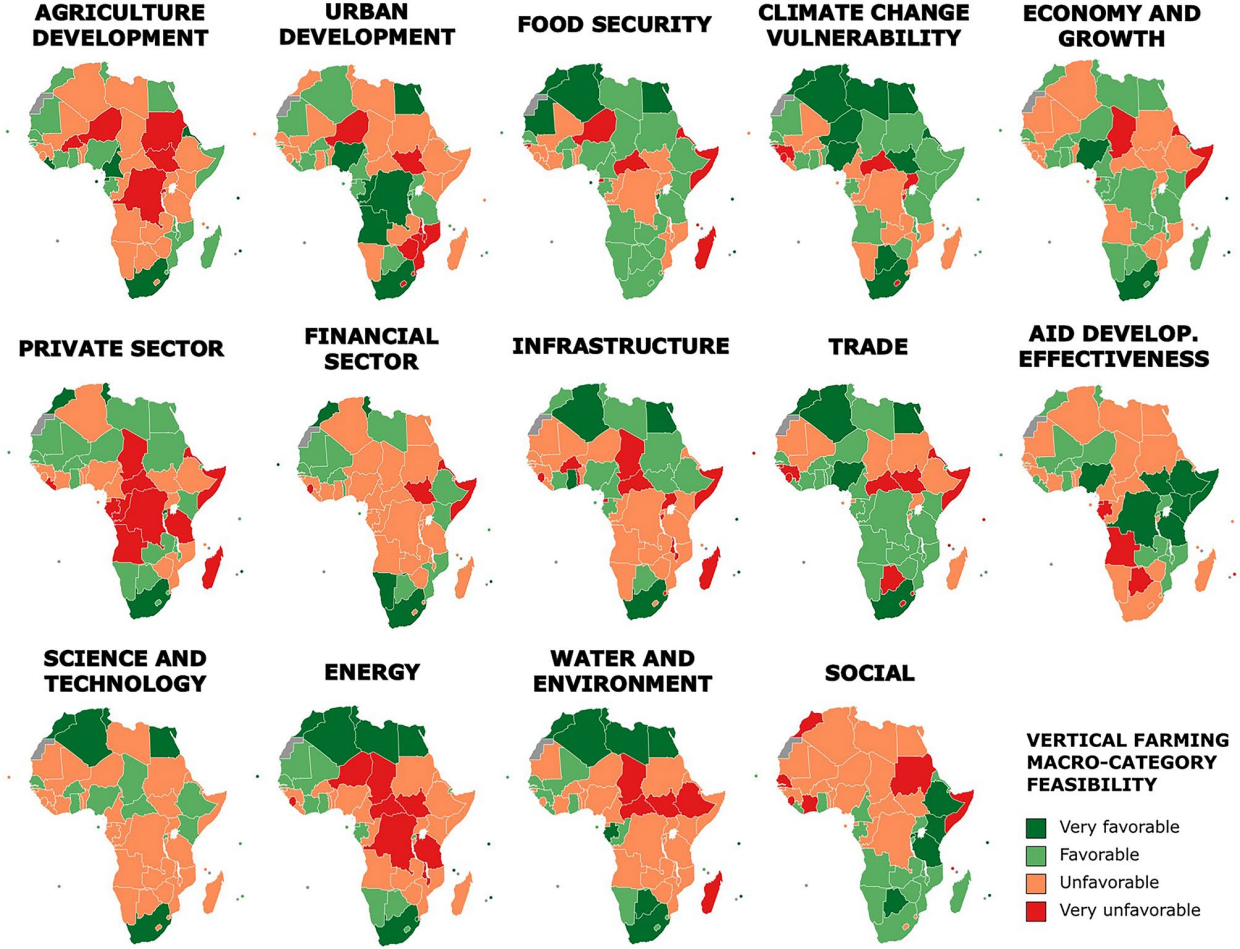
Categorization of vertical farming feasibility per each macro-category in African countries (generated with MapChart, https://www.mapchart.net/).

**Figure 4:**
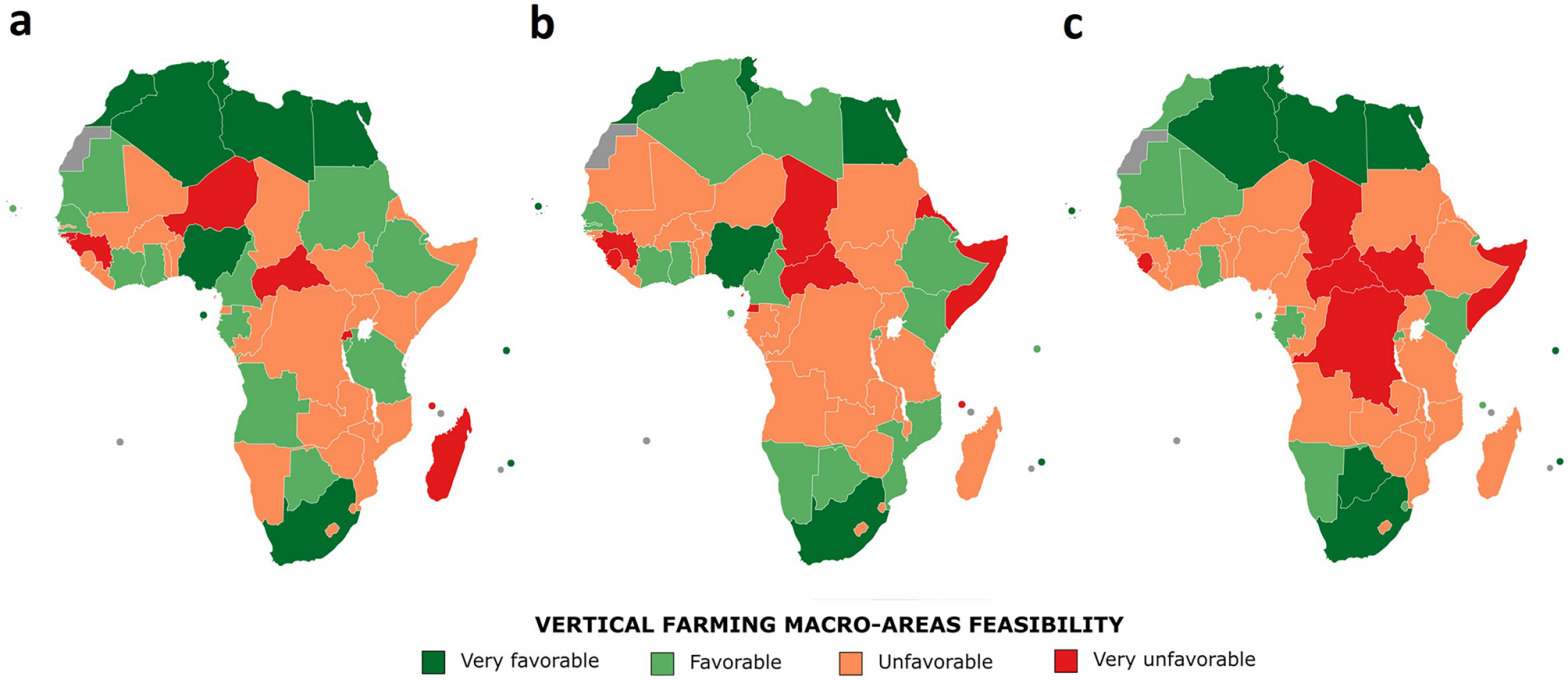
Categorization of vertical farming feasibility per macro-area in African countries. (**a**) Urban Agriculture Productivity Assurance and Food Security macro-area. Synthetic feasibility index (SFI) categorization as very favorable (> 0.519); favorable (0.519–0.419); unfavorable (0.419–0.318); very unfavorable (< 0.318). (**b**) Economic and Political Implications macro-area. Synthetic feasibility index (SFI) categorization as very favorable (> 0.380); favorable (0.380–0.290); unfavorable (0.290–0.200); very unfavorable (< 0.200). (**c**) Resources availability and Social Implication macro-area. Synthetic feasibility index (SFI) categorization as very favorable (> 0.589); favorable (0.589–0.460); unfavorable (0.460–0.331); very unfavorable (< 0.331) (generated with MapChart, https://www.mapchart.net/).

This study was performed following a methodological framework for the feasibility and sustainability assessment of vertical farming in Africa (Fig. 1, summarized in “Methods” section). The study considered 54 independent African Countries (Fig. 2), omitting Western Sahara as it has unknown international status^19^. From 147 indicators selected for this study, 14 macro-categories were elaborated to evaluate the feasibility of vertical farming in the African continent (Fig. 3, Table 1) which were grouped in 3 macro-areas (Fig. 4): (1) Urban Agriculture Productivity Assurance and Food Security (*Urban Development, Agriculture and Growth, Food Security, Climate Change Vulnerability*), (2) Economic and Political Implications (*Economy and Growth, Private Sector, Financial Sector, Infrastructure, Trade, Aid Development Effectiveness, Science and Technology*) and (3) Resources availability and Social Implication (*Energy, Water and Environment, Social*).

**Table 1:** Synthetic feasibility index (SFI) categorization per macro-category.

Synthetic feasibility index (SFI) categorization per macro-category				
Macro-categories	Very favorable	Favorable	Unfavorable	Very unfavorable
*Agriculture development*	> 0.530	0.530 to 0.450	0.450 to 0.371	< 0.371
*Urban development*	> 0.347	0.347 to 0.271	0.271 to 0.195	< 0.195
*Food security*	> 0.812	0.812 to 0.576	0.576 to 0.339	< 0.339
*Climate change vulnerability*	> 0.574	0.574 to 0.420	0.420 to 0.266	< 0.266
*Economy and growth*	> 0.470	0.470 to 0.399	0.399 to 0.328	< 0.328
*Private sector*	> 0.644	0.644 to 0.586	0.586 to 0.527	< 0.527
*Financial sector*	> 0.309	0.309 to 0.155	0.155 to 0.001	< 0.001
*Infrastructure*	> 0.548	0.548 to 0.316	0.316 to 0.084	< 0.084
*Trade*	> 0.439	0.439 to 0.270	0.270 to 0.100	< 0.100
*Aid development effectiveness*	> 0.374	0.374 to 0.246	0.246 to 0.118	< 0.118
*Science and technology*	> 0.214	0.214 to 0.085	0.085 to − 0.044	< − 0.044
*Energy*	> 0.732	0.732 to 0.519	0.519 to 0.305	< 0.305
*Water and environment*	> 0.547	0.547 to 0.402	0.402 to 0.258	< 0.258
*Social*	> 0.575	0.575 to 0.459	0.459 to 0.343	< 0.343

Among all macro-categories considered (Fig. 3), South Africa was classified as “very favorable” in 78.6% of the total, followed by Mauritius (64.3%), and Egypt and Morocco (57.1%). In contrast, Somalia and the Central Africa Republic were the countries more times categorized as “very unfavorable” (50%), followed by South Sudan and Sierra Leone (42.9%). In this regard, Somalia qualified as “very unfavorable” in macro-categories such as *Food Security*, *Economy and Growth*, *Private Sector*, *Finance Sector*, *Infrastructure*, *Trade* and *Social*. Moreover, *Food Security* and *Trade* were the macro-categories that showed to have more African countries in “very favorable” and “favorable” conditions (59.3 and 57.4%, respectively). In comparison, *Science and Technology* and *Financial Sector* accounted for a greater number of “unfavorable” and “very unfavorable” countries (68.5 and 66.7%, respectively). The highest average Synthetic Feasibility Index (SFI) was obtained by Seychelles, which showed a score close to 1 for *Energy* and *Food Security* (Supplementary Table 1). Additionally, *Science and Technology* and *Financial Sector* were the macro-categories in which there was a greater difference among countries with a coefficient of variation of 152 and 99.8%, respectively. On the other hand, *Private Sector* was highly uniform across African states with 9.9% of variation coefficient (Supplementary Table 2).

### Implications of vertical farming challenges

1. *Urban Agriculture Productivity Assurance and Food Security* According to their current trends, North African territory, islands such as Seychelles, Mauritius, Sao Tome and Principe and countries such as South Africa and Nigeria resulted in the best feasible areas for a safe urban agriculture production (“very favorable” categorization) (Fig. 4a, Supplementary Table 3). Due to higher *Urban development*, *Agricultural and Growth* and *Climate Change Vulnerability*, these countries are more feasible to implement indoor vertical farms in cities. Producing food in cities can enhance urban food security and healthy nutrition of its population, as products are expected to be sold locally, increasing the accessibility of fresh food^20^. In this macro-area, Egypt reported the greatest result (0.62 SFI) thanks to higher scores in terms of *Food Security* and *Urban Development* than South Africa, which had the second one (0.60 SFI). Moreover, islands such as Seychelles, Mauritius, and Sao Tome and Principe displayed scores among the best ones in terms of *Agriculture Development* and *Food Security*. North African countries resulted highly vulnerable to climate change, therefore, they will need to adapt their agriculture to face external environmental conditions. In terms of *Urban Development*, the highest score was obtained by Nigeria (Supplementary Table 1). Consequently, new ways of urban food production should be performed to avoid food insecurity in the future.
2. *Economic and Political Implications* The results showed a remarkable leading position of South Africa in this macro-area (0.69 SFI), followed by Morocco, Egypt, Tunisia, Mauritius, Nigeria and Cape Verde (Fig. 4b, Supplementary Table 3). Adequate and quality infrastructure and financial-sector development can be good policy options for transforming economic activities and sectors from traditional to modern. It is of strategic importance to maintain a policy and investment focus on agriculture. Enhancing the efficiency of public spending can be crucial to improve productivity and perform the agricultural transformation strategy. *Economy and Growth*, *Financial Sector*, *Trade*, and *Science and Technology* domains were led by South Africa. In this regard, South Africa resulted remarkably high in *Science and Technology* compared to the rest of the continent (Supplementary Table 1), being a policy that bets in *Science and Technology* necessary due to the scientific and technological requirements that are generated for the maintenance of an indoor vertical farm*.* Morocco and Seychelles showed the best score in *Private Sector* and *Infrastructure*, respectively (Supplementary Table 1). Investments from private and public sector are required due to the cost of implementing indoor vertical farms, that are particularly high. More attention should be paid to the private sector in terms of infrastructure upgrading and reforms to create a conducive business environment in order to bolster sustainable development.
3. *Resources availability and Social Implication* In this macro-area, Seychelles stands out with the highest index score (Fig. 4c, Supplementary Table 3) due to getting the best *Energy*, *Water and Environment* and *Social* results (Supplementary Table 1). Energy and water requirements, and high qualified labor force are necessary for vertical farming feasibility. Countries such as Mauritius, Tunisia, South Africa, Algeria, Libya, Egypt, Cape Verde and Botswana were classified as “very favorable” for this macro-area analysis (Fig. 4c, Supplementary Table 3). North African countries showed to be very favorable in *Energy* and *Water and Environment* macro-categories while in *Social* were categorized as unfavorable or very unfavorable. A low female labor force and scarce employment in relation to the population ratio were the main reasons for a negative *Social* categorization in these Northern countries. Botswana and Rwanda were highlighted due to being the second and third positions in *Social*, respectively (Supplementary Table 3). This high prevalence of employment (mostly in agriculture) is correlated with a high level of informality in work^21^. Consequently, indoor vertical farming industry can be an opportunity to the decent work deficits.

### Overall feasibility and sustainability assessment

**Figure 5:**
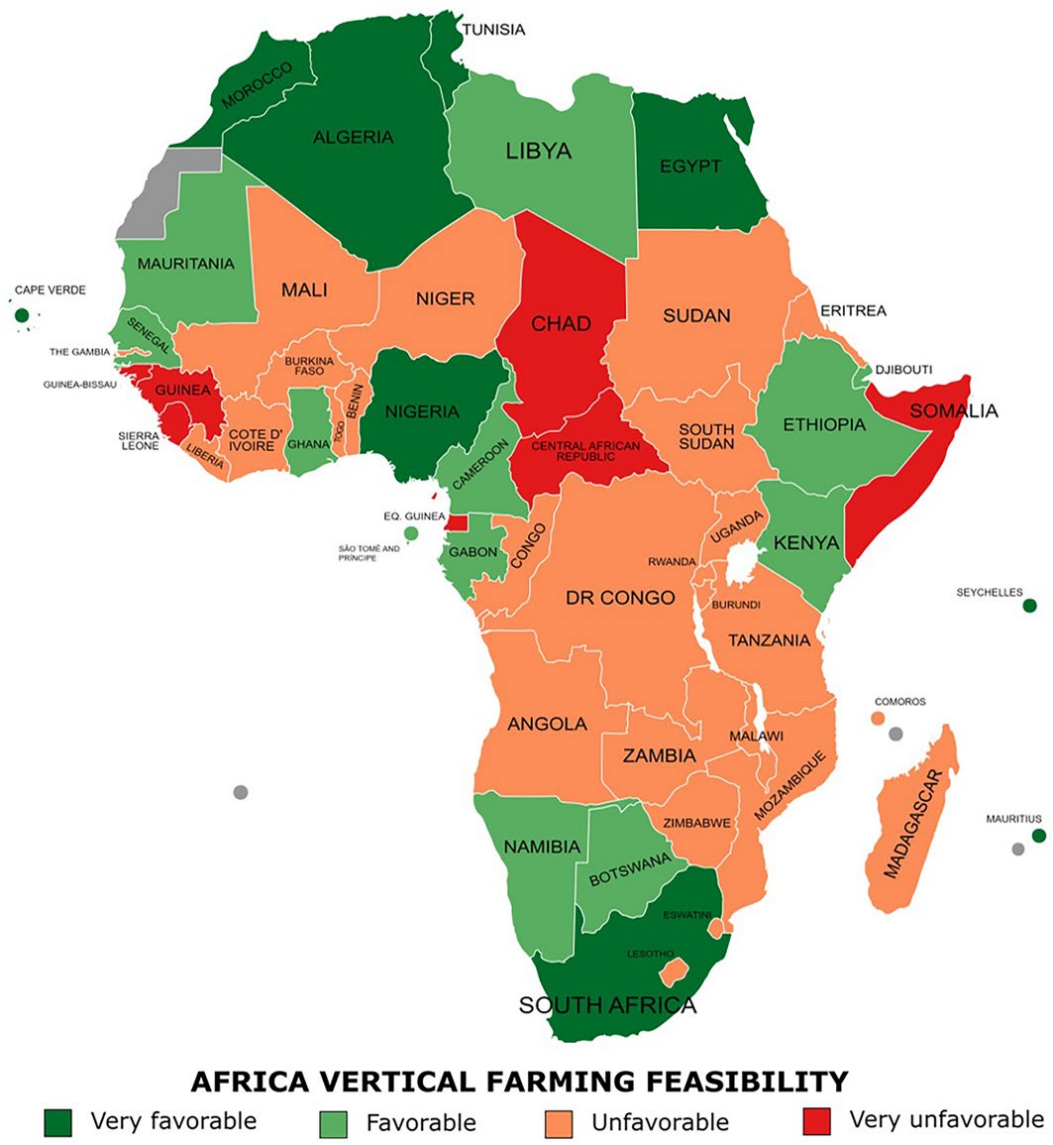
Categorization of African countries according to feasibility of vertical farming. Synthetic feasibility index (SFI) categorized in very favorable (> 0.453); favorable (0.453–0.363); unfavorable (0.363–0.273); very unfavorable (< 0.273) (generated with MapChart, https://www.mapchart.net/).

Considering the overall ranking of the feasibility of vertical farming in Africa, nine countries achieved a “very favorable” categorization (Fig. 5, Table 2). Most of the “very favorable” countries were from the North Africa region (Egypt, Morocco, Tunisia, and Algeria) but also small islands of the Atlantic (Cape Verde) and Pacific (Seychelles and Mauritius). However, a southern country, South Africa, resulted being the most favorable country (0.66 SFI). Besides, the only country from Middle West achieving a “very favorable” score was Nigeria. According to the UNSD 2022^22^ assignment of countries to geographical regions, most countries from West, Central, and East Africa subregions were reported to be “unfavorable” or “very unfavorable” for implementing indoor vertical farms. A poor economic growth, low private and financial sector presence, deficient energy (electrical) and water resources, inferior trade market, urban development and prevalence of food insecurity are some of the basis of this categorization. Sierra Leone, Somalia, and the Central African Republic showed the lowest scores. However, low technological, simplified and economically accessible solutions of vertical farming systems outdoors could be developed and adapted as new forms of agriculture according to the specific country situation. On the other hand, Northern and Southern Africa were the subregions more suitable for installing and operating an indoor vertical farm. The overall better results in the macro-areas analyzed highlighted a better subregional development, better conditions and more resources for this innovative farming method.

**Table 2:** Synthetic feasibility index (SFI), position and categorization for each African Country.

Country	Position	SFI	Categorization
Algeria	7	0.490	Very favorable
Angola	26	0.347	Unfavorable
Benin	36	0.321	Unfavorable
Botswana	12	0.422	Favorable
Burkina Faso	29	0.345	Unfavorable
Burundi	33	0.332	Unfavorable
Cape Verde	8	0.463	Very favorable
Cameroon	21	0.364	Favorable
Central African Republic	54	0.200	Very unfavorable
Chad	50	0.263	Very unfavorable
Comoros	47	0.278	Unfavorable
Congo, Dem. Rep	40	0.309	Unfavorable
Congo, Rep	38	0.314	Unfavorable
Cote d’Ivoire	22	0.358	Unfavorable
Djibouti	15	0.394	Favorable
Egypt	3	0.527	Very favorable
Equatorial Guinea	49	0.265	Very unfavorable
Eritrea	46	0.281	Unfavorable
Eswatini	31	0.338	Unfavorable
Ethiopia	19	0.379	Favorable
Gabon	16	0.391	Favorable
Gambia, The	23	0.355	Unfavorable
Ghana	11	0.423	Favorable
Guinea	48	0.273	Very unfavorable
Guinea-Bissau	51	0.254	Very unfavorable
Kenya	17	0.385	Favorable
Lesotho	39	0.312	Unfavorable
Liberia	27	0.347	Unfavorable
Libya	10	0.441	Favorable
Madagascar	45	0.286	Unfavorable
Malawi	37	0.316	Unfavorable
Mali	30	0.344	Unfavorable
Mauritania	18	0.382	Favorable
Mauritius	4	0.519	Very favorable
Morocco	5	0.517	Very favorable
Mozambique	35	0.331	Unfavorable
Namibia	20	0.373	Favorable
Niger	43	0.300	Unfavorable
Nigeria	9	0.455	Very favorable
Rwanda	24	0.349	Unfavorable
Sao Tome and Principe	13	0.409	Favorable
Senegal	14	0.399	favorable
Seychelles	2	0.553	Very favorable
Sierra Leone	52	0.242	Very unfavorable
Somalia	53	0.236	Very unfavorable
South Africa	1	0.661	Very favorable
South Sudan	44	0.291	Unfavorable
Sudan	34	0.332	Unfavorable
Tanzania	25	0.348	Unfavorable
Togo	42	0.301	Unfavorable
Tunisia	6	0.512	Very favorable
Uganda	41	0.305	Unfavorable
Zambia	32	0.338	Unfavorable
Zimbabwe	28	0.346	Unfavorable

Synthetic feasibility index categorized in very favorable (> 0.453); favorable (0.453–0.363); unfavorable (0.363–0.273); very unfavorable (< 0.273).

**Figure 6:**
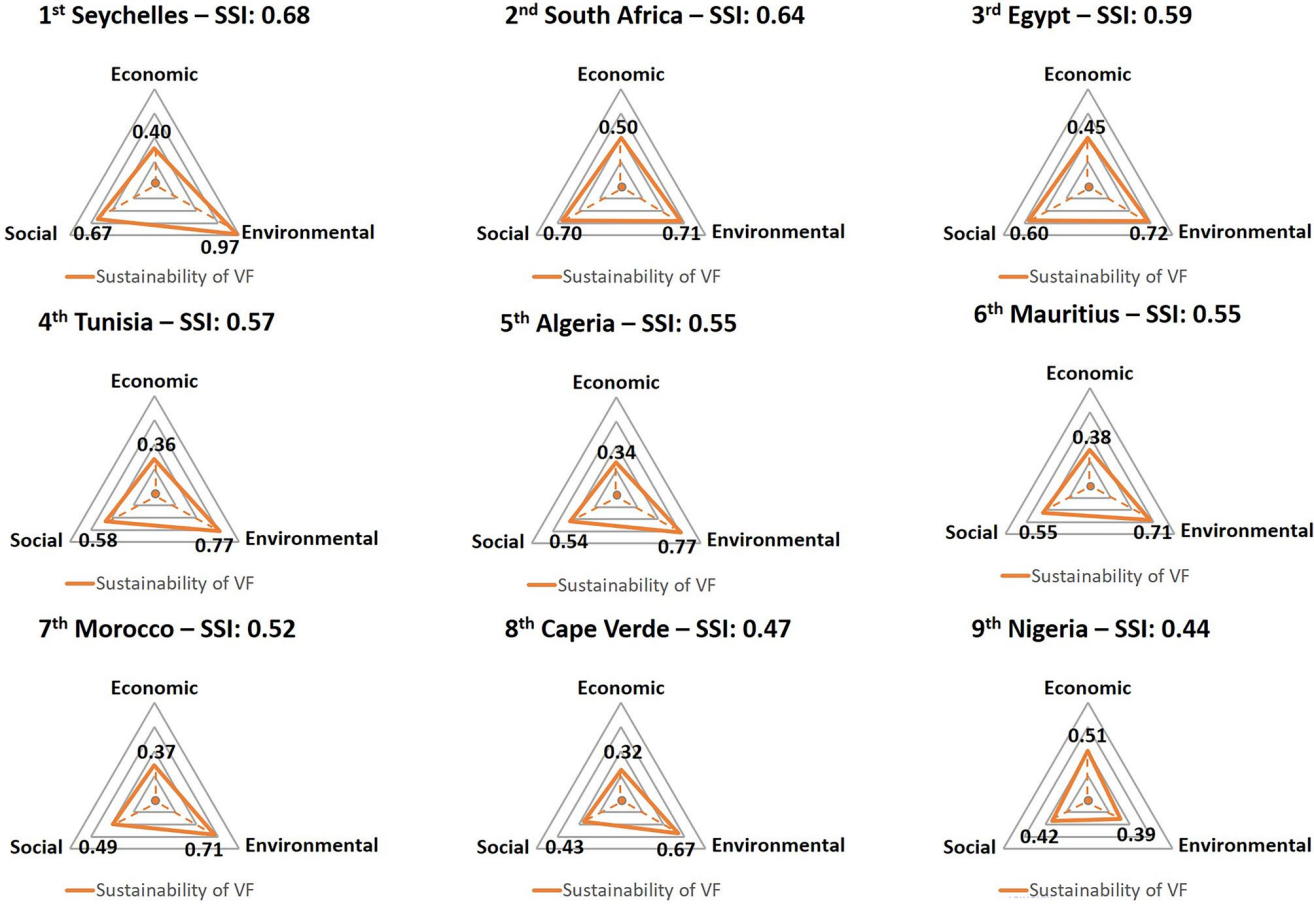
Assessment of the economic, social and environmental sustainability of vertical farming for African countries ranked as 'very favorable' in the feasibility classification. Final Synthetic Sustainability Index (SSI) calculated as average SFI of Economic (*Economy & Growth*, *Urban Development*), Environmental (*Energy*, *Water and Environment*) and Social (*Food Security*, *Social* and *Science and Technology*).

A final table was drawn up to assess the sustainability of all 54 African countries (Table 3), and final considerations were made for the 9 most feasible countries for vertical farming (named “very favorable”) (Fig. 6). Seychelles resulted to be a country where the operation of a vertical farm could be more sustainable (0.68 SSI), getting the best score in the Environmental domain and being the second in Social sustainability. Afterward, South Africa and Egypt showed to be the second and third countries, respectively. Both countries were among the best three countries in Economic and Social sustainability. North African countries (Egypt, Tunisia, Algeria, and Morocco) were highlighted to obtain higher Environmental sustainability outcomes. Moreover, Nigeria resulted the country where indoor vertical farms could be more economically sustainable due to increasing urban development and economic growth.

**Table 3:** Synthetic Sustainability Index (SSI), position and categorization of each African Country.

Country	Position	SSI	Categorization
Algeria	5	0.549	Very favorable
Angola	23	0.381	Unfavorable
Benin	39	0.320	Unfavorable
Botswana	10	0.458	Favorable
Burkina Faso	24	0.378	Unfavorable
Burundi	18	0.409	Favorable
Cape Verde	9	0.473	Favorable
Cameroon	27	0.373	Unfavorable
Central African Republic	53	0.208	Very unfavorable
Chad	46	0.281	Unfavorable
Comoros	38	0.323	Unfavorable
Congo. Dem. Rep	44	0.308	Unfavorable
Congo. Rep	35	0.343	Unfavorable
Cote d’Ivoire	34	0.344	Unfavorable
Djibouti	16	0.421	Favorable
Egypt	3	0.592	Very favorable
Equatorial Guinea	48	0.262	Very unfavorable
Eritrea	51	0.235	Very unfavorable
Eswatini	17	0.409	Favorable
Ethiopia	26	0.374	Unfavorable
Gabon	11	0.456	Favorable
Gambia, The	25	0.374	Unfavorable
Ghana	15	0.425	Favorable
Guinea	42	0.312	Unfavorable
Guinea-Bissau	49	0.253	Very unfavorable
Kenya	21	0.393	Favorable
Lesotho	29	0.364	Unfavorable
Liberia	33	0.346	Unfavorable
Libya	8	0.484	Favorable
Madagascar	45	0.302	Unfavorable
Malawi	40	0.318	Unfavorable
Mali	22	0.385	Favorable
Mauritania	14	0.426	Favorable
Mauritius	6	0.546	Very favorable
Morocco	7	0.521	Very favorable
Mozambique	43	0.309	Unfavorable
Namibia	20	0.399	Favorable
Niger	50	0.236	Very unfavorable
Nigeria	13	0.435	Favorable
Rwanda	28	0.365	Unfavorable
Sao Tome and Principe	12	0.451	Favorable
Senegal	19	0.406	Favorable
Seychelles	1	0.678	Very favorable
Sierra Leone	47	0.262	Very unfavorable
Somalia	54	0.184	Very unfavorable
South Africa	2	0.643	Very favorable
South Sudan	52	0.235	Very unfavorable
Sudan	30	0.361	Unfavorable
Tanzania	36	0.343	Unfavorable
Togo	41	0.314	Unfavorable
Tunisia	4	0.571	Very favorable
Uganda	37	0.334	Unfavorable
Zambia	32	0.353	Unfavorable
Zimbabwe	31	0.359	Unfavorable

Synthetic sustainability index categorized in very favorable (> 0.487); favorable (0.487–0.381); unfavorable (0.381–0.275); very unfavorable (< 0.275).

## Discussion

### The challenge of indoor vertical farms to ensure urban agriculture productivity and food security in Africa

North African territory, islands such as Seychelles, Mauritius, Sao Tome and Principe and countries such as South Africa and Nigeria showed remarkable potential in (1) *Urban Agriculture Productivity Assurance and Food Security* macro-area (Fig. 4a, Supplementary Table 3). Abdillahin and Sezgin^23^ highlighted that South Africa is leading the way toward a new future of urban food production in the African continent. Furthermore, islands such as Seychelles and Mauritius obtained the best scores in *Agriculture Development* and *Food security* (Fig. 3, Supplementary Table 1), countries that are lacking agricultural land and dependent on food imports due to their island status^24^. Therefore, indoor vertical farming industry could significantly impact the variability of cultivated crops grown and supplying them local and fresh produce. Moreover, North African countries are highly vulnerable to climate change (Fig. 3). In this regard, researchers increased their attention for the Northern Africa region as a “climate change hotspot”, with scenarios that predict an average rise in annual temperatures, more intense and longer-lasting droughts, and drops ranging 4 to 27% in the annual rainfall^9,25^. North African countries will need to adapt their agricultural development to face the future environmental challenges and especially water resource stresses^25^. In this framework, indoor vertical farming systems implementation can be an opportunity, enabling to reduce water use up to 95% as compared to open-field farming^26^.

In terms of *Urban Development*, the highest score was obtained by Nigeria, which has the highest urban population in Africa, with 107 million urban inhabitants in 2020, more than double compared with the second country on the list, Egypt (Supplementary Table 1)^27^. Consequently, as urban development projections progress, the country will face severe challenges in feeding its population. A study by Rahmann et al.^28^ who projected different scenarios of agricultural land needed by 2100 according to population growth, showed that Nigeria is not big enough to reach self-sufficiency in food production even if yields were increased dramatically. Indoor vertical farms can exponentially reduce the agricultural pressure on land, as cultivation is performed vertically (e.g., shelves, towers…), and the use of soil is not needed by adopting soilless cultivation methods^26^.

### Political and economic implications for the development of vertical farming in Africa

African economies are transforming rapidly. It is of strategic importance to maintain a policy and investment focus on agriculture, even as agriculture diminishes relative importance to the broader economy^29^. In this study, the (2) *Economic and Political Implications* macro-area was highlighted in countries like South Africa, Morocco, Egypt, Tunisia, Mauritius, Nigeria, Cape Verde and Seychelles (Fig. 4b, Supplementary Table 3). Economic stability directly impacts growth and development; however, investments in research and technology innovation are vital for boosting the economic growth in these countries^30^. South Africa showed a remarkable investment in *Science and Technology* macro-category (Supplementary Table 1). Besides, South Africa has implemented several policy initiatives that target agriculture as one of the critical sectors requiring modernization to support growth and development^31^. Northern African countries such as Egypt, Tunisia, Algeria, and Morocco also resulted “very favorable” (Fig. 3). Many countries have put ambitious national agricultural investment plans under the Comprehensive African Agriculture Development Programme (CAADP) that would increase agriculture growth sustainably and efficiently while reducing poverty^32^.

Vertical farms are usually building-integrated systems with an elevated initial cost due to the required infrastructures and its technological equipment and energy inputs^33^. Therefore, initial funding and private sector analysis are often required for these businesses to start, as modern agriculture in African regions is largely market-oriented and, therefore, dependent on the private sector^34^. South Africa, Mauritius, Morocco, and Tunisia resulted to be “very favorable” in both *Financial* and *Private Sector* macro-categories (Fig. 3). Private investments are dominant in scale and scope in the African agriculture sector (70%); however, 94% of the research and development is covered by public funds, suggesting a lack of attraction for private sector investment to increase innovations in agricultural research^35^. On the other hand, poor infrastructure has been highlighted as a major barrier to adopting improved technologies in agriculture^36^. Therefore, a well-industrialized economy is expected to have adequate infrastructure. Seychelles became the leading country in this *Infrastructure* macro-category, followed by South Africa, Cape Verde, Mauritius, Ghana, Algeria, and Egypt (Fig. 3, Supplementary Table 1).

### Resources availability and social implication of vertical farming in Africa

In terms of (3) *Resources availability and Social Implication*, countries such as Seychelles, Mauritius, Tunisia, South Africa, Algeria, Libya, Egypt, Cape Verde and Botswana were classified as “very favorable” (Fig. 4c). Energy requirements and, more specifically, access to electricity represent an essential input for indoor vertical farming implementation^33^. Proper artificial lighting, temperature, fans and ventilators, and heat and water pumps are some of the equipment that generally runs a vertical farm, requiring energy^37^. Accordingly, this study showed Seychelles as the best country in the *Energy* macro-category with a synthetic value of 1 (Supplementary Table 1). The global access deficit to electricity is centered in Sub-Saharan Africa, with the area accounting for the 75% of the World’s population without electricity access^38^. Additionally, water quantity and quality play an important role in environmental sustainability. Its availability is crucial for plant growth in vertical farming systems^39,40^. Countries such as Seychelles, Tunisia, Algeria, Morocco, Libya, Egypt, Mauritius, South Africa, Botswana and Gabon resulted “very favorable” into the *Water and Environment* macro-category (Fig. 3). Fischer et al.^41^ estimated that the most significant increases in irrigation water requirements are projected to occur in Africa (+ 300%) from 2000 to 2080, resulting in the most critical values of annual renewable freshwater resources. Moreover, by 2050, the available water per capita per year will drop to a critical value below 1000 m^3^ in the North African belt and eastern and southern Africa, and the Middle East^42^. Consequently, in the future, there will also be the need to produce food with less water, allowing for the uptake of indoor vertical farming systems which highly optimize the water use^43^.

Successful agricultural production is increasingly knowledge-intensive. The agricultural workforce will need to enhance productivity, deal with future food challenges, and take advantage from innovative emerging opportunities for promoting inclusive forms of agricultural productivity growth^44^. Countries such as Seychelles, Botswana, Rwanda, Mauritius, Kenya, Ethiopia, and Tanzania showed to be “very favorable” in the *Social* macro-category (Fig. 3). For instance, Rwanda is in the process of structural transformation, which has involved workers and capital in progressively higher productivity activities, resulting in rapid technological changes^45^.

### Sustainability at the edge: opening up the feasibility of vertical farming in Africa

This study evaluates the overall feasibility of several macro-categories representing different domains. Nine African countries were “very favorable” for operating indoor vertical farms, being the ones with the highest synthetic feasibility index (SFI): South Africa (0.66 SFI), islands such as Seychelles (0.55 SFI), Mauritius (0.52 SFI) and Cape Verde (0.46 SFI), Northern Africa region (Egypt, Morocco, Tunisia, Algeria, with 0.53, 0.52, 0.51 and 0.49 SFI, respectively) and Nigeria (0.46 SFI) (Fig. 5, Table 2). In these countries, a trend to innovate and improve the current agriculture systems with indoor vertical farms could be possible. With financial and technological support from governments and the private sector attraction to city dwellers and companies, the uptake of indoor vertical farms in Africa can be achieved. Countries that reached lower feasibility scores can opt for simplified vertical cultivation solutions adapted to the economic and social conditions and the locally available materials. Borgwardt and Endress^46^ developed a case study to implement a vertical farm in the Maun Science Park in Botswana (0.42 SFI; “favorable”), showing that conditions for sustainable food security through this cultivation system can be achieved with the ongoing development of the country. However, they also recognized that vertical farming applications and market scope would be limited compared to other continents, such as Europe. Furthermore, sustainability of resources and safety in the food production line is a significant issue globally that can make vertical farming a powerful way of cultivation through technological applications and soilless cultivation systems^47^. Africa must move from a scarcely productive farming system towards sustainable intensification and eco-friendliness^48^. In this study, Seychelles, as first, followed by South Africa and Egypt, were the three countries where the implementation of vertical farming systems could be more sustainable (Fig. 6, Table 3).

From the Environmental analysis of sustainability, Seychelles, Tunisia and Algeria showed higher synthetic values (Fig. 6). These three countries have programs to implement more renewable energies. For instance, Tunisia and Algeria have set a target of 30% and 27% of renewable energy within their energy mix by 2030, focusing mainly on increasing solar and wind generation^24,49,50^. This renewable energy supply can enable more sustainable food production through indoor vertical agriculture while increasing its market potential^51^. Building a vertical farm requires a high initial investment (from the private sector or public programs), and production costs are higher than traditional farming^52^. Therefore, countries with good economic growth, capital generation, and proper urban development will be more suitable for sustainably implementing vertical farming in their cities. Among the nine most feasible countries, Nigeria, South Africa, and Egypt obtained the highest values in the Economic analysis for sustainability (Fig. 6). Vertical farming can create jobs in several sectors of engineering, biotechnology, construction, and research and development^16^. In this study, South Africa, Seychelles, and Egypt showed the best sustainability Social analysis scores (Fig. 6). Seychelles is one of the African countries where most of its labor force is with advanced education^27^, which is advantageous for the broad spectrum of job descriptions typically observed in a vertical farm. Locating food production in the African city centers is expected to increase economic growth in the area^53^.

### Future perspectives

Countries such as South Africa, Seychelles, Egypt, Mauritius, Morocco, Tunisia, Algeria, Cape Verde, and Nigeria showed the best prospective for indoor vertical farming implementation. These are African countries where the integration of vertical farming into the urban areas may help reduce poverty, contribute to food safety and increase its contextual sustainability. However, its competitiveness in the local market could remain unclear with current providers still capable to sell cheaper products. In this regard, the indicator Consumer Price Food Index (% of price change of the average food basket purchased by households) showed that among the sustainably “very favorable” countries, Egypt and Nigeria are experiencing the higher inflation rates in the food basket while the others resulted to have a more stable trend^54^.

Nevertheless, to enhance the environmental sustainability and improve the efficiency and sufficiency of food production supplies in African society, it is necessary to develop diverse and influential vertical cultivation systems adapted to the reality of each country. The least feasible countries can opt for modular and low-cost innovative solutions of vertical farming systems which can offer new opportunities for young or small-scale growers without being dependent on the high investments typical of high tech indoor vertical farms^55^. Moreover, exploiting urban water resources, harvesting the rainwater or reusing the building's thermal mass^56,57^ are solutions other than those mentioned in the study, that could however lead to a greater sustainability of indoor vertical cultivations.

## Methods

Indicators represent quantitative tools that synthesize and simplify the data relevant to the assessment of specific phenomena. They are helpful for communication, evaluation and facilitate strategic decisions making. It can be assumed that indicators remain one of the primary instruments for monitoring sustainable development as they present this concept rationally and measurably. In the same way, for this study, indicators can be defined as a statistical measure informing about the feasibility and the sustainability in the social, environmental, and economic spheres^58^. For example, Erol et al.^59^ conducted a feasibility assessment for blockchain applications in different industries by developing a list of helpful feasibility indicators. High-quality feasibility indicators should enable decision-makers to take informed decisions when developing business information technology strategies. The quality of an indicator can be assessed through six aspects: underlying data, suitability, measurability, representation of the case at hand, accuracy, and communicability to stakeholders. However, developing a complete list of indicators that can cover all these attributes may not always be possible; therefore, a more practical list is often created^59^. In developing such a list for vertical farming feasibility assessment, the critical task is to determine whether this technology is technically, strategically, and financially viable. The sustainability assessment is then carried on solely for the countries where the technology is feasible, tailoring the three pillars of sustainability, namely environmental, economic, and social, to the vertical farming technology. Two research questions were defined for this study:

1. How feasible is vertical farming with artificial lighting in the African countries?
2. How sustainable is vertical farming with artificial lighting for the countries that can implement this technology?

### Hypothesis and limitations

Assessing vertical farming feasibility and sustainability can require multidimensionality^60^. With the introduction of sustainable development as a concept in the 80s, different criteria and indicators have been selected to evaluate the sustainability of agriculture^61^. Consequently, there is no long-standing consensus on which indicators are more suitable or relevant to assess feasibility or sustainability^62^. Moreover, there are no frameworks for vertical farming implementation in a country. Therefore, there can be conceptual and methodological limitations. In this paper, the matrix was based on the macro-categories created for feasibility assessment, adapting them to the main dimensions of the vertical farming sector. Therefore, for this study, the sustainable assessment can be defined as a statistical measure informing the sustainability of vertical farming implementation in the African countries where it is most feasible. Furthermore, four more thematic areas (*Urban development*, *Energy*, *Food Security*, and *Science and Technology*) were added to the three main pillars of sustainability (Economic, Environmental and Social)^63^ according to their crucial role in ensuring vertical farming sustainability.

The indicators include both stimulating and destimulating factors in the economic, social and environmental spheres. The central hypothesis of the study is that a change of the synthetic indicator, averaged from proposed indicators, leads to a change in the level of feasibility and sustainability potential of vertical farming^64^. This hypothesis lays the foundation for the study of the influence of contributing factors, here named “macro-categories”, for the construction of the algorithm, for future analysis in different territorial contexts (geographically and dimensions wise) and consequent decision making.

To substantiate the proposed hypothesis and achieve the set goal, the following steps are proposed, both for the feasibility and sustainability assessment (Fig. 1):

1. Identification of suitable indicators based on technical, strategical, and financial requirements for vertical farming feasibility and sustainability in the African continent.
2. Retrieving the chosen data from the World Bank database in order to be gathered in observational matrices.
3. Determination of the main factors, here named “macro-categories”, suitable for hosting all the chosen indicators. An indicator can be placed in more than one macro-category, potentially increasing or decreasing its influence on the final scoring, depending on how the indicator is further classified.
4. Classification of all the chosen indicators in relation to the macro-category/ies they belong. They can be classified as stimulant, destimulant, or nominant^65^.
5. Normalization procedure through the Zero Unitarization Method (also known as Min-Max Normalization) according to how the indicator was classified^58,66^.
6. Construction of a Synthetic Feasibility Index and a Synthetic Sustainability Index for each of the 54 African Countries by averaging the 14 Synthetic Indexes corresponding to each macro-category.
7. Feasibility and sustainability classification of the 54 African Countries based on a statistical comparison of the final Synthetic Indexes^58,67^.

The main limitations of this study lie in:

- The level of definition of data on a geographical scale, as an analysis by country, that does not capture differences among vast portions of territory within national borders.
- The lack of data for some of the countries and indicators analyzed.
- The choice of the last five years of available data for each country as the reference time for the assessment, due to the greater availability of data and the intention to maximize the sample's representativeness.

### Method of choice for indicators

Defining quantitative objectives depends on the nature of each variable. The following variables can be distinguished: stimulants, destimulants and nominants. Although many variables could be smoothly classified as stimulant and destimulant, none of them could be described as nominant (values that have their optimal in the interval between the minimum and the maximum). Consequently, there were no nominant variables in the indicators set as all the variables that have been processed in the present study were solely classified as stimulant or destimulant. It is paramount to stress that the same variable could be classified as stimulant or destimulant depending on its macro-category^65^.

### Classification of indicators into macro-categories

The main aim was to construct a synthetic index to assess vertical farming feasibility and sustainability in African countries. This measure encompasses the multiple factors influencing vertical farming suitability in three main areas, economic, social, and environmental. Specific indicators of development from the World Bank and FAO have been gathered in influence matrices and then selected for the chosen macro-categories (Supplementary Tables 5, 6). The macro-categories for the feasibility analysis were grouped in 3 macro-areas (Fig. 4) for interpretation of results and were the following: (1) *Urban Agriculture Productivity Assurance and Food Security* (*Urban Development*, *Agriculture and Growth*, *Food Security* and *Climate Change Vulnerability*), (2) *Economic and Political Implications* (*Economy and Growth*, *Private Sector*, *Financial Sector*, *Infrastructure*, *Trade*, *Aid Development Effectiveness*, *Science and Technology*) and (3) *Resources availability and Social Implication* (*Energy*, *Water and Environment*, *Social*). It was decided to perform two separate analyses, one for feasibility and a subsequent one for sustainability, corresponding to the two research questions stated above. The sustainability analysis considered the 3 main pillars of sustainability (Economical, Social and Environmental)^63^. Furthermore, 4 more thematic areas were added to the 3 domains previously mentioned (*Urban development* as Economic, *Energy* as Environmental, *Food Security* and *Science and Technology* as Social) due to their importance in vertical farming sustainability. Both feasibility and sustainability analysis were based on the same procedure, which constructed a final synthetic index for each country. It should also be noted that by opting to include some indicators in more than one macro-category, it was decided to place more weight on these specific indicators concerning the final result of the analysis.

### Construction of the synthetic index

The synthetic index was constructed using an average and standard deviation linear ordering method. Its construction procedure is carried out based on the following consecutive stages:

1. Selecting diagnostic features (indicators) and determining the nature of variables to each macro-category: stimulant and destimulant^65^.
2. For indicators’ comparability, the normalization of diagnostic features was carried out using a zero-unitarization procedure (or Min–Max Normalization) based on the following formula^58,66^:
  For stimulant variables:
  $${n}_{i}=\frac{{x}_{i}-{x}_{min}}{{x}_{max}-{x}_{min}}.$$
  For destimulant variables:
  $${n}_{i}=\frac{{x}_{max}-{x}_{i}}{{x}_{max}-{x}_{min}},$$
  where x_i_ is the variable’s value at the i-year, while x_max_ and x_min_ are the maximum and minimum values among all 54 countries for the same i-year. This approach scales the data into different ranges based on the minimum and maximum values, with the benefit that boundaries can be set and all indicators have an identical interval (0, 1). However, it is worth stressing that normalized values do not preserve proportionality and reflect the percentage of the range of maximum and minimum. Indeed, if the maximum and minimum are outliers, the range between the two heavily influences the final result. It is also important to highlight that the variance difference is not completely eliminated^68^. Nonetheless, this technique is suitable for the task and is widely applied in constructing several composite indicators, such as the human development index (HDI)^69^.
  The next step was to calculate an average normalized value n_av_ for each country. Normalization was carried out for a matrix covering the data from 2006 to 2020. As previously stated in the limitation section, given the higher data availability for the most recent years and wanting to consider the most recent trends, we only considered the last five years of available data. Working with a time span rather than only the most recent values allowed for defining a common development pattern. After applying zero unitarization, the variable was measured on an interval scale with zero minimum^58,70^.
3. The following reasoning was applied to determine the object-pattern coordinates. In the case of stimulants, maximum values were considered the most favorable values of diagnostic characteristics. Conversely, in the case of destimulants, minimum values were considered the most favorable values of diagnostic characteristics. The most favorable indicator values of the chosen 5-year time frame make up the object pattern^58,67^.
4. It was calculated a synthetic index x_j_ for each country (j) by dividing all the n_av_ of each selected variable by the number of variables. Therefore, we obtained one value for each country, also called the synthetic index. Based on the synthetic variable’s arithmetic mean ($\overline{{\text{x}}}$) and standard deviation (S_x_) the following four groups were identified^58,67^:
  Very favorable: x_j_ ≥ $$\overline{{\text{x}}}$$ + S_x_.
  Favorable: $$\overline{{\text{x}}}$$ < x_j_ < $$\overline{{\text{x}}}$$ + S_x_.
  Unfavorable: $$\overline{{\text{x}}}$$ − S_x_ ≤ x_j_ < $$\overline{{\text{x}}}$$.
  Very unfavorable: x_j_ < $$\overline{{\text{x}}}$$ − S_x_.

## Supplementary Information

Supplementary Tables.

## Data Availability

Data of World Bank Development Indicators are available at https://databank.worldbank.org/. Data of Development Indicators from Food and Agriculture Organization of the United Nations (FAO) are available at https://www.fao.org/faostat/en/#data. Source data are provided with this paper.
