# Supplementary material for: A methodological tool for sustainability and feasibility assessment of indoor vertical farming with artificial lighting in Africa
Source: Sci Rep. 2023 Feb 6;13:2109. doi: 10.1038/s41598-023-29027-8 (PMC9902558; doi:10.1038/s41598-023-29027-8)
Supplement: Supplementary file 1 — Supplementary Tables. [file 41598_2023_29027_MOESM1_ESM.docx]

Supplementary material

**Supplementary Table 1.** Results of average Synthetic Feasibility Index (SFI) in every African country per macro-category considered.

| SYNTHETIC FEASIBILITY INDEX (SFI) PER MACRO-CATEGORY | | | | | | | | | | | | | | | | | | | | | | | | | | | |
| --- | --- | --- | --- | --- | --- | --- | --- | --- | --- | --- | --- | --- | --- | --- | --- | --- | --- | --- | --- | --- | --- | --- | --- | --- | --- | --- | --- |
| Country | **Economy and growth** | **Agriculture Development** | | **Private Sector** | | **Energy** | | **Financial Sector** | | **Infrastructure** | | **Trade** | | **Water and Environment** | | **Climate Change Vulnerability** | | **Science & Technology** | | **Aid Develop. Effectiveness** | | **Social** | | **Urban Development** | | **Food Security** | |
| Algeria | 0.398 | 0.441 | 0.570 | | 0.875 | | 0.280 | | 0.598 | | 0.490 | | 0.659 | | 0.688 | | 0.246 | | 0.127 | | 0.442 | | 0.288 | | 0.935 | |  |
| Angola | 0.364 | 0.431 | 0.523 | | 0.394 | | 0.102 | | 0.302 | | 0.348 | | 0.279 | | 0.434 | | 0.067 | | 0.060 | | 0.538 | | 0.356 | | 0.668 | |  |
| Benin | 0.385 | 0.385 | 0.550 | | 0.363 | | 0.100 | | 0.137 | | 0.270 | | 0.334 | | 0.566 | | 0.017 | | 0.235 | | 0.447 | | 0.213 | | 0.484 | |  |
| Botswana | 0.463 | 0.426 | 0.638 | | 0.614 | | 0.112 | | 0.528 | | 0.069 | | 0.554 | | 0.675 | | 0.016 | | 0.086 | | 0.650 | | 0.320 | | 0.592 | |  |
| Burkina Faso | 0.401 | 0.316 | 0.628 | | 0.485 | | 0.223 | | 0.065 | | 0.311 | | 0.276 | | 0.379 | | 0.138 | | 0.258 | | 0.379 | | 0.285 | | 0.686 | |  |
| Burundi | 0.359 | 0.423 | 0.634 | | 0.334 | | 0.132 | | 0.007 | | 0.251 | | 0.366 | | 0.103 | | 0.049 | | 0.238 | | 0.516 | | 0.413 | | 0.824 | |  |
| Cape Verde | 0.406 | 0.468 | 0.636 | | 0.873 | | 0.585 | | 0.859 | | 0.058 | | 0.476 | | 0.317 | | 0.018 | | 0.251 | | 0.485 | | 0.245 | | 0.807 | |  |
| Cameroon | 0.413 | 0.531 | 0.567 | | 0.470 | | 0.091 | | 0.393 | | 0.319 | | 0.324 | | 0.346 | | 0.041 | | 0.239 | | 0.486 | | 0.286 | | 0.589 | |  |
| Central African Republic | 0.334 | 0.397 | 0.523 | | 0.080 | | 0.061 | | 0.049 | | 0.032 | | 0.166 | | 0.069 | | 0.142 | | 0.219 | | 0.412 | | 0.226 | | 0.097 | |  |
| Chad | 0.265 | 0.403 | 0.492 | | 0.264 | | 0.039 | | 0.050 | | 0.132 | | 0.187 | | 0.426 | | 0.099 | | 0.172 | | 0.356 | | 0.198 | | 0.601 | |  |
| Comoros | 0.393 | 0.424 | 0.537 | | 0.816 | | 0.070 | | 0.119 | | 0.044 | | 0.448 | | 0.276 | | 0.028 | | 0.168 | | 0.291 | | 0.282 | | 0.000 | |  |
| Congo, Dem. Rep. | 0.412 | 0.370 | 0.484 | | 0.178 | | 0.007 | | 0.306 | | 0.301 | | 0.305 | | 0.292 | | 0.064 | | 0.403 | | 0.427 | | 0.394 | | 0.380 | |  |
| Congo, Rep. | 0.402 | 0.430 | 0.510 | | 0.335 | | 0.119 | | 0.156 | | 0.298 | | 0.482 | | 0.334 | | 0.037 | | 0.143 | | 0.425 | | 0.405 | | 0.318 | |  |
| Cote d'Ivoire | 0.429 | 0.492 | 0.587 | | 0.521 | | 0.081 | | 0.372 | | 0.398 | | 0.377 | | 0.499 | | 0.061 | | 0.172 | | 0.322 | | 0.291 | | 0.406 | |  |
| Djibouti | 0.446 | 0.489 | 0.639 | | 0.574 | | 0.152 | | 0.282 | | 0.320 | | 0.373 | | 0.492 | | 0.000 | | 0.193 | | 0.488 | | 0.317 | | 0.750 | |  |
| Egypt | 0.433 | 0.505 | 0.604 | | 0.849 | | 0.150 | | 0.579 | | 0.597 | | 0.587 | | 0.618 | | 0.475 | | 0.187 | | 0.443 | | 0.464 | | 0.891 | |  |
| Equatorial Guinea | 0.309 | 0.586 | 0.569 | | 0.523 | | 0.089 | | 0.000 | | 0.136 | | 0.319 | | 0.437 | | 0.000 | | 0.051 | | 0.392 | | 0.291 | | 0.000 | |  |
| Eritrea | 0.140 | 0.565 | 0.522 | | 0.458 | | 0.000 | | 0.294 | | 0.091 | | 0.294 | | 0.668 | | 0.001 | | 0.147 | | 0.522 | | 0.231 | | 0.000 | |  |
| Eswatini | 0.446 | 0.508 | 0.622 | | 0.749 | | 0.082 | | 0.000 | | 0.081 | | 0.502 | | 0.371 | | 0.053 | | 0.210 | | 0.436 | | 0.110 | | 0.568 | |  |
| Ethiopia | 0.391 | 0.441 | 0.584 | | 0.418 | | 0.216 | | 0.355 | | 0.165 | | 0.257 | | 0.384 | | 0.101 | | 0.538 | | 0.588 | | 0.218 | | 0.644 | |  |
| Gabon | 0.417 | 0.490 | 0.522 | | 0.683 | | 0.071 | | 0.400 | | 0.305 | | 0.550 | | 0.429 | | 0.005 | | 0.074 | | 0.464 | | 0.299 | | 0.771 | |  |
| Gambia, The | 0.357 | 0.399 | 0.557 | | 0.511 | | 0.009 | | 0.530 | | 0.300 | | 0.431 | | 0.298 | | 0.051 | | 0.257 | | 0.408 | | 0.290 | | 0.569 | |  |
| Ghana | 0.424 | 0.494 | 0.609 | | 0.580 | | 0.040 | | 0.671 | | 0.434 | | 0.438 | | 0.416 | | 0.096 | | 0.285 | | 0.475 | | 0.267 | | 0.691 | |  |
| Guinea | 0.389 | 0.432 | 0.569 | | 0.478 | | 0.039 | | 0.085 | | 0.095 | | 0.416 | | 0.245 | | 0.005 | | 0.174 | | 0.343 | | 0.227 | | 0.322 | |  |
| Guinea-Bissau | 0.391 | 0.454 | 0.537 | | 0.375 | | 0.074 | | 0.285 | | 0.080 | | 0.296 | | 0.234 | | 0.000 | | 0.123 | | 0.422 | | 0.286 | | 0.000 | |  |
| Kenya | 0.407 | 0.436 | 0.631 | | 0.478 | | 0.170 | | 0.253 | | 0.339 | | 0.359 | | 0.391 | | 0.145 | | 0.424 | | 0.591 | | 0.235 | | 0.535 | |  |
| Lesotho | 0.424 | 0.424 | 0.633 | | 0.521 | | 0.146 | | 0.282 | | 0.015 | | 0.412 | | 0.191 | | 0.002 | | 0.136 | | 0.442 | | 0.175 | | 0.570 | |  |
| Liberia | 0.371 | 0.582 | 0.521 | | 0.337 | | 0.104 | | 0.239 | | 0.377 | | 0.500 | | 0.323 | | 0.001 | | 0.297 | | 0.542 | | 0.297 | | 0.371 | |  |
| Libya | 0.457 | 0.445 | 0.600 | | 0.909 | | 0.165 | | 0.395 | | 0.384 | | 0.597 | | 0.634 | | 0.014 | | 0.164 | | 0.400 | | 0.234 | | 0.778 | |  |
| Madagascar | 0.408 | 0.490 | 0.487 | | 0.491 | | 0.079 | | 0.040 | | 0.270 | | 0.211 | | 0.300 | | 0.011 | | 0.219 | | 0.533 | | 0.234 | | 0.229 | |  |
| Malawi | 0.421 | 0.460 | 0.618 | | 0.285 | | 0.039 | | 0.076 | | 0.300 | | 0.395 | | 0.388 | | 0.040 | | 0.313 | | 0.564 | | 0.190 | | 0.330 | |  |
| Mali | 0.395 | 0.394 | 0.633 | | 0.542 | | 0.203 | | 0.107 | | 0.187 | | 0.463 | | 0.294 | | 0.065 | | 0.299 | | 0.455 | | 0.240 | | 0.531 | |  |
| Mauritania | 0.392 | 0.455 | 0.612 | | 0.707 | | 0.165 | | 0.309 | | 0.179 | | 0.380 | | 0.442 | | 0.009 | | 0.200 | | 0.361 | | 0.296 | | 0.836 | |  |
| Mauritius | 0.475 | 0.551 | 0.712 | | 0.847 | | 0.481 | | 0.763 | | 0.275 | | 0.568 | | 0.569 | | 0.117 | | 0.087 | | 0.592 | | 0.278 | | 0.947 | |  |
| Morocco | 0.468 | 0.455 | 0.725 | | 0.804 | | 0.443 | | 0.468 | | 0.535 | | 0.621 | | 0.606 | | 0.240 | | 0.361 | | 0.334 | | 0.270 | | 0.911 | |  |
| Mozambique | 0.386 | 0.451 | 0.591 | | 0.330 | | 0.193 | | 0.250 | | 0.293 | | 0.329 | | 0.355 | | 0.057 | | 0.331 | | 0.509 | | 0.186 | | 0.367 | |  |
| Namibia | 0.436 | 0.413 | 0.628 | | 0.551 | | 0.314 | | 0.315 | | 0.302 | | 0.472 | | 0.318 | | 0.073 | | 0.139 | | 0.488 | | 0.223 | | 0.553 | |  |
| Niger | 0.406 | 0.298 | 0.610 | | 0.303 | | 0.055 | | 0.194 | | 0.322 | | 0.294 | | 0.706 | | 0.058 | | 0.362 | | 0.410 | | 0.178 | | 0.000 | |  |
| Nigeria | 0.537 | 0.479 | 0.587 | | 0.422 | | 0.050 | | 0.469 | | 0.558 | | 0.352 | | 0.631 | | 0.119 | | 0.554 | | 0.420 | | 0.475 | | 0.720 | |  |
| Rwanda | 0.384 | 0.358 | 0.699 | | 0.531 | | 0.104 | | 0.202 | | 0.356 | | 0.292 | | 0.297 | | 0.099 | | 0.311 | | 0.642 | | 0.304 | | 0.307 | |  |
| Sao Tome and Principe | 0.388 | 0.641 | 0.584 | | 0.679 | | 0.170 | | 0.435 | | 0.128 | | 0.407 | | 0.375 | | 0.148 | | 0.239 | | 0.376 | | 0.328 | | 0.828 | |  |
| Senegal | 0.412 | 0.489 | 0.597 | | 0.555 | | 0.239 | | 0.288 | | 0.325 | | 0.470 | | 0.564 | | 0.153 | | 0.240 | | 0.328 | | 0.269 | | 0.659 | |  |
| Seychelles | 0.552 | 0.650 | 0.610 | | 1.000 | | 0.202 | | 0.970 | | 0.046 | | 0.944 | | 0.397 | | 0.086 | | 0.127 | | 0.923 | | 0.243 | | 0.998 | |  |
| Sierra Leone | 0.370 | 0.446 | 0.539 | | 0.297 | | 0.000 | | 0.060 | | 0.015 | | 0.280 | | 0.234 | | 0.002 | | 0.251 | | 0.285 | | 0.217 | | 0.386 | |  |
| Somalia | 0.179 | 0.513 | 0.437 | | 0.313 | | 0.000 | | 0.012 | | 0.097 | | 0.272 | | 0.413 | | 0.000 | | 0.550 | | 0.150 | | 0.224 | | 0.150 | |  |
| South Africa | 0.576 | 0.563 | 0.673 | | 0.856 | | 0.818 | | 0.921 | | 0.914 | | 0.566 | | 0.669 | | 0.763 | | 0.193 | | 0.560 | | 0.423 | | 0.759 | |  |
| South Sudan | 0.338 | 0.238 | 0.540 | | 0.199 | | 0.000 | | 0.367 | | 0.000 | | 0.221 | | 0.650 | | 0.036 | | 0.629 | | 0.421 | | 0.106 | | 0.324 | |  |
| Sudan | 0.337 | 0.295 | 0.631 | | 0.493 | | 0.031 | | 0.361 | | 0.194 | | 0.333 | | 0.425 | | 0.053 | | 0.183 | | 0.329 | | 0.247 | | 0.734 | |  |
| Tanzania | 0.406 | 0.441 | 0.526 | | 0.303 | | 0.046 | | 0.203 | | 0.324 | | 0.267 | | 0.505 | | 0.025 | | 0.425 | | 0.582 | | 0.281 | | 0.534 | |  |
| Togo | 0.407 | 0.378 | 0.570 | | 0.421 | | 0.228 | | 0.074 | | 0.286 | | 0.320 | | 0.322 | | 0.045 | | 0.160 | | 0.379 | | 0.292 | | 0.335 | |  |
| Tunisia | 0.467 | 0.473 | 0.644 | | 0.856 | | 0.359 | | 0.417 | | 0.421 | | 0.687 | | 0.598 | | 0.338 | | 0.265 | | 0.456 | | 0.255 | | 0.937 | |  |
| Uganda | 0.385 | 0.390 | 0.573 | | 0.417 | | 0.074 | | 0.025 | | 0.269 | | 0.262 | | 0.260 | | 0.041 | | 0.337 | | 0.511 | | 0.297 | | 0.427 | |  |
| Zambia | 0.404 | 0.400 | 0.598 | | 0.334 | | 0.063 | | 0.239 | | 0.341 | | 0.382 | | 0.326 | | 0.030 | | 0.287 | | 0.488 | | 0.266 | | 0.569 | |  |
| Zimbabwe | 0.383 | 0.421 | 0.595 | | 0.426 | | 0.053 | | 0.263 | | 0.310 | | 0.382 | | 0.490 | | 0.024 | | 0.192 | | 0.561 | | 0.159 | | 0.578 | |  |

**Supplementary Table 2.** Synthetic measures for the distance between countries among macro-categories considered for assessing feasibility.

| SYNTHETIC MEASURES PER MACRO-CATEGORY | | | | | | | | | | | | | | |  |
| --- | --- | --- | --- | --- | --- | --- | --- | --- | --- | --- | --- | --- | --- | --- | --- |
| Specification | **Economy and growth** | **Agriculture Development** | **Private Sector** | **Energy** | **Financial Sector** | **Infrastructure** | **Trade** | **Water and Environment** | **Climate Change Vulnerability** | **Science & Technology** | **Aid Develop. Effectiveness** | **Social** | **Urban Development** | **Food Security** | |
| Median | 0.403 | 0.443 | 0.589 | 0.488 | 0.104 | 0.283 | 0.298 | 0.379 | 0.394 | 0.050 | 0.227 | 0.445 | 0.270 | 0.574 | |
| Min. value | 0.140 | 0.238 | 0.437 | 0.080 | 0.000 | 0.000 | 0.015 | 0.166 | 0.069 | 0.000 | 0.051 | 0.150 | 0.106 | 0.000 | |
| Max. value | 0.576 | 0.650 | 0.725 | 1.000 | 0.818 | 0.970 | 0.914 | 0.944 | 0.706 | 0.706 | 0.629 | 0.923 | 0.475 | 0.998 | |
| Difference quotient | 0.435 | 0.412 | 0.289 | 0.920 | 0.818 | 0.970 | 0.899 | 0.778 | 0.637 | 0.706 | 0.577 | 0.773 | 0.369 | 0.998 | |
| Coefficient of variation (%) | 17.816 | 17.639 | 9.972 | 41.168 | 99.283 | 73.522 | 62.743 | 35.832 | 36.643 | 150.961 | 51.937 | 25.364 | 28.052 | 41.503 | |
| Standard deviation | 0.071 | 0.079 | 0.058 | 0.214 | 0.155 | 0.232 | 0.169 | 0.144 | 0.154 | 0.129 | 0.128 | 0.116 | 0.076 | 0.237 | |
| Arithmetic mean | 0.399 | 0.450 | 0.586 | 0.519 | 0.154 | 0.316 | 0.270 | 0.402 | 0.420 | 0.085 | 0.246 | 0.459 | 0.271 | 0.576 | |

**Supplementary Table 3.** Results of average Synthetic Feasibility Index (SFI) in every African country per macro-area considered.

| SYNTHETIC FEASIBILITY INDEX (SFI) PER MACRO-AREA | | | | | | |
| --- | --- | --- | --- | --- | --- | --- |
| Country | **Agriculture urban productivity and Food security** | **Categorization** | **Economic and Political implications** | **Categorization** | **Resource availability and social implication** | **Categorization** |
| Algeria | 0.588 | very favorable | 0.362 | favorable | 0.659 | very favorable |
| Angola | 0.472 | favorable | 0.252 | unfavorable | 0.404 | unfavorable |
| Benin | 0.412 | unfavorable | 0.244 | unfavorable | 0.381 | unfavorable |
| Botswana | 0.503 | favorable | 0.297 | favorable | 0.606 | very favorable |
| Burkina Faso | 0.416 | unfavorable | 0.289 | unfavorable | 0.380 | unfavorable |
| Burundi | 0.441 | favorable | 0.239 | unfavorable | 0.405 | unfavorable |
| Cape Verde | 0.459 | favorable | 0.402 | very favorable | 0.611 | very favorable |
| Cameroon | 0.438 | favorable | 0.295 | favorable | 0.427 | unfavorable |
| Central African Republic | 0.197 | very unfavorable | 0.194 | very unfavorable | 0.219 | very unfavorable |
| Chad | 0.407 | unfavorable | 0.179 | very unfavorable | 0.269 | very unfavorable |
| Comoros | 0.245 | very unfavorable | 0.194 | very unfavorable | 0.518 | favorable |
| Congo, Dem. Rep. | 0.359 | unfavorable | 0.282 | unfavorable | 0.303 | very unfavorable |
| Congo, Rep. | 0.371 | unfavorable | 0.238 | unfavorable | 0.414 | unfavorable |
| Cote d'Ivoire | 0.422 | favorable | 0.300 | favorable | 0.406 | unfavorable |
| Djibouti | 0.512 | favorable | 0.290 | favorable | 0.478 | favorable |
| Egypt | 0.620 | very favorable | 0.432 | very favorable | 0.626 | very favorable |
| Equatorial Guinea | 0.329 | unfavorable | 0.165 | very unfavorable | 0.411 | unfavorable |
| Eritrea | 0.366 | unfavorable | 0.171 | very unfavorable | 0.424 | unfavorable |
| Eswatini | 0.389 | unfavorable | 0.213 | unfavorable | 0.562 | favorable |
| Ethiopia | 0.422 | favorable | 0.336 | favorable | 0.421 | unfavorable |
| Gabon | 0.497 | favorable | 0.256 | unfavorable | 0.566 | favorable |
| Gambia, The | 0.389 | unfavorable | 0.295 | favorable | 0.450 | unfavorable |
| Ghana | 0.467 | favorable | 0.366 | favorable | 0.498 | favorable |
| Guinea | 0.307 | very unfavorable | 0.194 | very unfavorable | 0.412 | unfavorable |
| Guinea-Bissau | 0.243 | very unfavorable | 0.213 | unfavorable | 0.364 | unfavorable |
| Kenya | 0.399 | unfavorable | 0.338 | favorable | 0.476 | favorable |
| Lesotho | 0.340 | unfavorable | 0.234 | unfavorable | 0.458 | unfavorable |
| Liberia | 0.393 | unfavorable | 0.273 | unfavorable | 0.460 | unfavorable |
| Libya | 0.523 | very favorable | 0.311 | favorable | 0.636 | very favorable |
| Madagascar | 0.313 | very unfavorable | 0.216 | unfavorable | 0.412 | unfavorable |
| Malawi | 0.342 | unfavorable | 0.258 | unfavorable | 0.415 | unfavorable |
| Mali | 0.365 | unfavorable | 0.270 | unfavorable | 0.487 | favorable |
| Mauritania | 0.507 | favorable | 0.267 | unfavorable | 0.483 | favorable |
| Mauritius | 0.586 | very favorable | 0.416 | very favorable | 0.669 | very favorable |
| Morocco | 0.560 | very favorable | 0.463 | very favorable | 0.587 | favorable |
| Mozambique | 0.340 | unfavorable | 0.300 | favorable | 0.389 | unfavorable |
| Namibia | 0.377 | unfavorable | 0.315 | favorable | 0.504 | favorable |
| Niger | 0.295 | very unfavorable | 0.287 | unfavorable | 0.336 | unfavorable |
| Nigeria | 0.576 | very favorable | 0.411 | very favorable | 0.398 | unfavorable |
| Rwanda | 0.317 | very unfavorable | 0.308 | favorable | 0.488 | favorable |
| Sao Tome and Principe | 0.543 | very favorable | 0.299 | favorable | 0.487 | favorable |
| Senegal | 0.495 | favorable | 0.322 | favorable | 0.451 | unfavorable |
| Seychelles | 0.572 | very favorable | 0.370 | favorable | 0.956 | very favorable |
| Sierra Leone | 0.321 | unfavorable | 0.177 | very unfavorable | 0.287 | very unfavorable |
| Somalia | 0.325 | unfavorable | 0.182 | very unfavorable | 0.245 | very unfavorable |
| South Africa | 0.603 | very favorable | 0.694 | very favorable | 0.661 | very favorable |
| South Sudan | 0.330 | unfavorable | 0.273 | unfavorable | 0.280 | very unfavorable |
| Sudan | 0.425 | favorable | 0.256 | unfavorable | 0.385 | unfavorable |
| Tanzania | 0.440 | favorable | 0.279 | unfavorable | 0.384 | unfavorable |
| Togo | 0.332 | unfavorable | 0.253 | unfavorable | 0.373 | unfavorable |
| Tunisia | 0.566 | very favorable | 0.416 | very favorable | 0.666 | very favorable |
| Uganda | 0.343 | unfavorable | 0.244 | unfavorable | 0.397 | unfavorable |
| Zambia | 0.391 | unfavorable | 0.280 | unfavorable | 0.401 | unfavorable |
| Zimbabwe | 0.412 | unfavorable | 0.260 | unfavorable | 0.456 | unfavorable |

**Supplementary Table 4.** Synthetic measures for the distance between countries among macro-areas considered for assessing feasibility.

| SYNTHETIC MEASURES PER MACRO-AREA | | | |
| --- | --- | --- | --- |
| Specification | **Agriculture urban productivity and Food security** | **Economic and Political implications** | **Resource availability and social implication** |
| Median | 0.409 | 0.280 | 0.426 |
| Min. value | 0.197 | 0.165 | 0.219 |
| Max. value | 0.620 | 0.694 | 0.956 |
| Difference quotient | 0.423 | 0.529 | 0.736 |
| Coefficient of variation (%) | 23.97 | 31.03 | 28.11 |
| Standard deviation | 0.100 | 0.090 | 0.129 |
| Arithmetic mean | 0.418 | 0.290 | 0.460 |

**Supplementary Table 5**. Macro-categories and single indicators used to build the Synthetic Feasibility Index (SFI).

|  | **Synthetic Feasibility Index (SFI)** |  |  |
| --- | --- | --- | --- |
| **Macro-categories** | **Indicators** | **Indicator Type** | **Time Period** |
| **Economy & Growth** | Adjusted net national income (current US$) | Stimulant | 2015-2019 |
|  | Adjusted net national income per capita (current US$) | Stimulant | 2015-2019 |
|  | Adjusted savings: energy depletion (% of GNI) | Destimulant | 2015-2019 |
|  | Adjusted savings: natural resources depletion (% of GNI) | Destimulant | 2015-2019 |
|  | Agriculture, forestry, and fishing, value added (annual % growth) | Stimulant | 2016-2020 |
|  | Consumer Prices, Food Indices (2015 = 100) | Destimulant | 2016-2020 |
|  | Foreign direct investment, net (BoP, current US$) | Stimulant | 2016-2020 |
|  | Foreign direct investment, net inflows (BoP, current US$) | Stimulant | 2015-2019 |
|  | GDP (current US$) | Stimulant | 2016-2020 |
|  | GDP per capita (current US$) | Stimulant | 2016-2020 |
|  | GNI (current US$) | Stimulant | 2016-2020 |
|  | GNI per capita, Atlas method (current US$) | Stimulant | 2016-2020 |
|  | Imports of goods and services (% of GDP) | Stimulant | 2016-2020 |
|  | Imports of goods and services (BoP, current US$) | Stimulant | 2016-2020 |
|  | Machinery and transport equipment (% of value added in manufacturing) | Stimulant | 2014-2018 |
|  | Manufactures imports (% of merchandise imports) | Stimulant | 2016-2020 |
|  | Manufacturing, value added (% of GDP) | Stimulant | 2016-2020 |
|  | Net capital account (BoP, current US$) | Stimulant | 2016-2020 |
|  | Net financial account (BoP, current US$) | Stimulant | 2016-2020 |
|  | Net primary income (BoP, current US$) | Stimulant | 2016-2020 |
|  | Net secondary income (BoP, current US$) | Stimulant | 2016-2020 |
|  | Net trade in goods (BoP, current US$) | Stimulant | 2016-2020 |
|  | Net trade in goods and services (BoP, current US$) | Stimulant | 2016-2020 |
| **Agriculture Development** | Agricultural land (% of land area) | Stimulant | 2014-2018 |
|  | Agricultural land (sq. km) | Stimulant | 2014-2018 |
|  | Agricultural methane emissions (% of total) | Destimulant | 2004-2008 |
|  | Agricultural nitrous oxide emissions (% of total) | Destimulant | 2004-2008 |
|  | Agricultural nitrous oxide emissions (% of total) | Destimulant | 2004-2008 |
|  | Annual freshwater withdrawals, agriculture (% of total freshwater withdrawal) | Stimulant | 2015-2019 |
|  | Arable land (% of land area) | Stimulant | 2014-2018 |
|  | Arable land (hectares per person) | Stimulant | 2014-2018 |
|  | Arable land (hectares) | Stimulant | 2014-2018 |
|  | Average precipitation in depth (mm per year) | Stimulant | 2016-2020 |
|  | Crop production index (2014-2016 = 100) | Stimulant | 2014-2018 |
|  | Fertilizer consumption (% of fertilizer production) | Stimulant | 2014-2018 |
|  | Fertilizer consumption (kilograms per hectare of arable land) | Stimulant | 2014-2018 |
|  | Rural land area (sq. km) | Stimulant | 2010* |
|  | Rural population | Stimulant | 2016-2020 |
|  | Rural population (% of total population) | Stimulant | 2016-2020 |
| **Private sector** | Cost of business start-up procedures (% of GNI per capita) | Destimulant | 2015-2019 |
|  | Cost to export, border compliance (US$) | Destimulant | 2015-2019 |
|  | Cost to import, border compliance (US$) | Destimulant | 2015-2019 |
|  | Ease of doing business score (0 = lowest performance to 100 = best performance) | Stimulant | 2015-2019 |
|  | Export unit value index (2000 = 100) | Stimulant | 2015-2019 |
|  | Firms experiencing electrical outages (% of firms) | Destimulant | 2006-2020* |
|  | Firms experiencing losses due to theft and vandalism (% of firms) | Destimulant | 2006-2020* |
|  | Firms formally registered when operations started (% of firms) | Stimulant | 2007-2020* |
|  | Firms that spend on R&D (% of firms) | Stimulant | 2011-2020* |
|  | Firms using banks to finance investment (% of firms) | Stimulant | 2006-2020* |
|  | Firms using banks to finance working capital (% of firms) | Stimulant | 2006-2020* |
|  | Import unit value index (2000 = 100) | Destimulant | 2015-2019 |
|  | Losses due to theft and vandalism (% of annual sales of affected firms) | Destimulant | 2006-2020* |
|  | Medium and high-tech manufacturing value added (% manufacturing value added) | Stimulant | 2015-2019 |
|  | Merchandise trade (% of GDP) | Stimulant | 2016-2020 |
|  | New business density (new registrations per 1,000 people ages 15-64) | Stimulant | 2014-2018 |
|  | New businesses registered (number) | Stimulant | 2014-2018 |
|  | Start-up procedures to register a business (number) | Destimulant | 2015-2019 |
|  | Time required to enforce a contract (days) | Destimulant | 2015-2019 |
|  | Time required to get electricity (days) | Destimulant | 2015-2019 |
|  | Time required to obtain an operating license (days) | Destimulant | 2006-2020* |
|  | Time required to register property (days) | Destimulant | 2015-2019 |
|  | Time required to start a business (days) | Destimulant | 2015-2019 |
|  | Time spent dealing with the requirements of government regulations (% of senior management time) | Destimulant | 2006-2020* |
|  | Time to obtain an electrical connection (days) | Destimulant | 2006-2020* |
| **Energy** | Access to electricity (% of population) | Stimulant | 2015-2019 |
|  | Access to electricity, rural (% of rural population) | Stimulant | 2015-2019 |
|  | Access to electricity, urban (% of urban population) | Stimulant | 2015-2019 |
|  | Energy use (kg of oil equivalent per capita) | Stimulant | 2010-2014 |
|  | Fossil fuel energy consumption (% of total) | Stimulant | 2010-2014 |
|  | Value lost due to electrical outages (% of sales for affected firms) | Destimulant | 2006-2020* |
| **Financial sector** | Domestic credit provided by financial sector (% of GDP) | Stimulant | 2006-2020* |
|  | Domestic credit to private sector (% of GDP) | Stimulant | 2016-2020 |
|  | Domestic credit to private sector by banks (% of GDP) | Stimulant | 2006-2020* |
|  | Market capitalization of listed domestic companies (% of GDP) | Stimulant | 2006-2020* |
|  | Market capitalization of listed domestic companies (current US$) | Stimulant | 2016-2020 |
| **Infrastructure** | Electric power consumption (kWh per capita) | Stimulant | 2010-2014 |
|  | Electric power transmission and distribution losses (% of output) | Destimulant | 2010-2014 |
|  | Individuals using the Internet (% of population) | Stimulant | 2016-2020 |
|  | Investment in energy with private participation (current US$) | Stimulant | 2016-2020 |
|  | Public private partnerships investment in energy (current US$) | Stimulant | 2016-2020 |
| **Trade** | Export unit value index (2000 = 100) | Stimulant | 2015-2019 |
|  | Exports of goods and services (current US$) | Stimulant | 2016-2020 |
|  | High-technology exports (current US$) | Stimulant | 2016-2020 |
|  | Imports of goods and services (current US$) | Stimulant | 2016-2020 |
|  | Lead time to export, median case (days) | Destimulant | 2014-2018 |
|  | Lead time to import, median case (days) | Destimulant | 2014-2018 |
|  | Merchandise exports (current US$) | Stimulant | 2016-2020 |
|  | Merchandise imports (current US$) | Stimulant | 2016-2020 |
| **Environment** | Level of water stress: freshwater withdrawal as a proportion of available freshwater resources | Stimulant | 2016-2020 |
|  | People using at least basic drinking water services (% of population) | Stimulant | 2016-2020 |
|  | People using at least basic drinking water services, rural (% of rural population) | Stimulant | 2016-2020 |
|  | People using at least basic drinking water services, urban (% of urban population) | Stimulant | 2016-2020 |
|  | People using safely managed drinking water services (% of population) | Stimulant | 2016-2020 |
|  | People using safely managed drinking water services, rural (% of rural population) | Stimulant | 2016-2020 |
|  | People using safely managed drinking water services, urban (% of urban population) | Stimulant | 2016-2020 |
|  | Plant species (higher), threatened | Destimulant | 2016-2020 |
|  | Renewable internal freshwater resources per capita (cubic meters) | Stimulant | 2016-2020 |
|  | Renewable internal freshwater resources, total (billion cubic meters) | Stimulant | 2016-2020 |
| **Climate change vulnerability** | Total greenhouse gas emissions (kt of CO2 equivalent) | Stimulant | 2014-2018 |
|  | Droughts, floods, extreme temperatures (% of population, average 1990-2009) | Stimulant | 1990-2009* |
|  | Electricity production from coal sources (% of total) | Stimulant | 2011-2015 |
|  | Electricity production from hydroelectric sources (% of total) | Destimulant | 2011-2015 |
|  | Electricity production from natural gas sources (% of total) | Destimulant | 2010-2014 |
|  | Electricity production from oil, gas and coal sources (% of total) | Stimulant | 2011-2015 |
|  | Electricity production from renewable sources, excluding hydroelectric (kWh) | Destimulant | 2011-2015 |
|  | Renewable electricity output (% of total electricity output) | Destimulant | 2011-2015 |
|  | Renewable energy consumption (% of total final energy consumption) | Destimulant | 2014-2018 |
|  | Population, total | Stimulant | 2016-2020 |
|  | Population in urban agglomerations of more than 1 million (% of total population) | Stimulant | 2016-2020 |
| **Science & Technology** | Charges for the use of intellectual property, payments (BoP, current US$) | Stimulant | 2016-2020 |
|  | Charges for the use of intellectual property, receipts (BoP, current US$) | Stimulant | 2016-2020 |
|  | High-technology exports (current US$) | Stimulant | 2016-2020 |
|  | Patent applications, residents | Stimulant | 2015-2019 |
|  | Patent applications, nonresidents | Stimulant | 2015-2019 |
|  | Research and development expenditure (% of GDP) | Stimulant | 2014-2018 |
|  | Researchers in R&D (per million people) | Stimulant | 2014-2018 |
|  | Scientific and technical journal articles | Stimulant | 2014-2018 |
|  | Technicians in R&D (per million people) | Stimulant | 2014-2018 |
| **Aid development Effectiveness** | Grants, excluding technical cooperation (BoP, current US$) | Stimulant | 2017* |
|  | Net ODA received per capita (current US$) | Stimulant | 2017* |
|  | Net ODA received (% of central government expense) | Stimulant | 2017* |
|  | Net ODA received (% of imports of goods, services and primary income) | Stimulant | 2017* |
|  | Net official development assistance received (current US$) | Stimulant | 2017* |
|  | Net official flows from UN agencies, FAO (current US$) | Stimulant | 2017* |
|  | Technical cooperation grants (BoP, current US$) | Stimulant | 2017* |
| **Social** | Children in employment, total (% of children ages 7-14 | Destimulant | 2011-2015 |
|  | Employment to population ratio, 15+, total (%) (modeled ILO estimate) | Stimulant | 2016-2020 |
|  | GDP per person employed (constant 2017 PPP $) | Stimulant | 2016-2020 |
|  | Labor force participation rate, female (% of female population ages 15+) (modeled ILO estimate) | Stimulant | 2015-2019 |
|  | Labor force participation rate, male (% of male population ages 15+) (modeled ILO estimate) | Stimulant | 2015-2019 |
|  | Labor force participation rate, total (% of total population ages 15+) (modeled ILO estimate) | Stimulant | 2016-2020 |
|  | Labor force with advanced education (% of total working-age population with advanced education) | Stimulant | 2015-2019 |
|  | Labor force, female (% of total labor force) | Stimulant | 2015-2019 |
|  | Labor force, total | Stimulant | 2016-2020 |
|  | Unemployment, total (% of total labor force) (modeled ILO estimate) | Destimulant | 2016-2020 |
|  | Wage and salaried workers, female (% of female employment) (modeled ILO estimate) | Stimulant | 2015-2019 |
|  | Wage and salaried workers, male (% of male employment) (modeled ILO estimate) | Stimulant | 2015-2019 |
|  | Wage and salaried workers, total (% of total employment) (modeled ILO estimate) | Stimulant | 2015-2019 |
| **Urban development** | Population density (people per sq. km of land area) | Stimulant | 2016-2020 |
|  | Population in largest city | Stimulant | 2016-2020 |
|  | Population in the largest city (% of urban population) | Stimulant | 2016-2020 |
|  | Population in urban agglomerations of more than 1 million (% of total population) | Stimulant | 2016-2020 |
|  | Urban land area (sq. km) | Stimulant | 2010* |
|  | Urban population | Stimulant | 2016-2020 |
|  | Urban population (% of total population) | Stimulant | 2016-2020 |
|  | Urban population growth (annual %) | Stimulant | 2016-2020 |
| **Food security** | Prevalence of moderate or severe food insecurity in the population (%) | Destimulant | 2015-2019 |
|  | Prevalence of severe food insecurity in the population (%) | Destimulant | 2015-2019 |
|  | Prevalence of undernourishment (% of population) | Destimulant | 2015-2019 |
|  | Use of insecticide-treated bed nets (% of under-5 population) | Destimulant | 2015-2019 |
|  |  |  |  |

* Indicators with data available for a specific year or arranged as data covering a long time period

**Supplementary Table 6**. Macro-categories and single indicators of development used to build the Synthetic Sustainability Index (SSI).

|  |  | **Synthetic Sustainability Index (SSI)** |  |  |
| --- | --- | --- | --- | --- |
|  | **Macro-categories** | **Indicators** | **Indicator Type** | **Time Period** |
| **ENVIRONMENTAL SUSTAINABILITY** | **Energy** | Access to electricity (% of population) | Stimulant | 2015-2019 |
|  |  | Access to electricity, rural (% of rural population) | Stimulant | 2015-2019 |
|  |  | Access to electricity, urban (% of urban population) | Stimulant | 2015-2019 |
|  |  | Energy use (kg of oil equivalent per capita) | Stimulant | 2010-2014 |
|  |  | Fossil fuel energy consumption (% of total) | Stimulant | 2010-2014 |
|  |  | Value lost due to electrical outages (% of sales for affected firms) | Destimulant | 2006-2020* |
|  | **Environment** | Level of water stress: freshwater withdrawal as a proportion of available freshwater resources | Stimulant | 2016-2020 |
|  |  | People using at least basic drinking water services (% of population) | Stimulant | 2016-2020 |
|  |  | People using at least basic drinking water services, rural (% of rural population) | Stimulant | 2016-2020 |
|  |  | People using at least basic drinking water services, urban (% of urban population) | Stimulant | 2016-2020 |
|  |  | People using safely managed drinking water services (% of population) | Stimulant | 2016-2020 |
|  |  | People using safely managed drinking water services, rural (% of rural population) | Stimulant | 2016-2020 |
|  |  | People using safely managed drinking water services, urban (% of urban population) | Stimulant | 2016-2020 |
|  |  | Plant species (higher), threatened | Destimulant | 2016-2020 |
|  |  | Renewable internal freshwater resources per capita (cubic meters) | Stimulant | 2016-2020 |
|  |  | Renewable internal freshwater resources, total (billion cubic meters) | Stimulant | 2016-2020 |
| **ECONOMIC SUSTAINABILTY** | **Economy & Growth** | Adjusted net national income (current US$) | Stimulant | 2015-2019 |
|  |  | Adjusted net national income per capita (current US$) | Stimulant | 2015-2019 |
|  |  | Adjusted savings: energy depletion (% of GNI) | Destimulant | 2015-2019 |
|  |  | Adjusted savings: natural resources depletion (% of GNI) | Destimulant | 2015-2019 |
|  |  | Agriculture, forestry, and fishing, value added (annual % growth) | Stimulant | 2016-2020 |
|  |  | Consumer Prices, Food Indices (2015 = 100) | Destimulant | 2016-2020 |
|  |  | Foreign direct investment, net (BoP, current US$) | Stimulant | 2016-2020 |
|  |  | Foreign direct investment, net inflows (BoP, current US$) | Stimulant | 2015-2019 |
|  |  | GDP (current US$) | Stimulant | 2016-2020 |
|  |  | GDP per capita (current US$) | Stimulant | 2016-2020 |
|  |  | GNI (current US$) | Stimulant | 2016-2020 |
|  |  | GNI per capita, Atlas method (current US$) | Stimulant | 2016-2020 |
|  |  | Imports of goods and services (% of GDP) | Stimulant | 2016-2020 |
|  |  | Imports of goods and services (BoP, current US$) | Stimulant | 2016-2020 |
|  |  | Machinery and transport equipment (% of value added in manufacturing) | Stimulant | 2014-2018 |
|  |  | Manufactures imports (% of merchandise imports) | Stimulant | 2016-2020 |
|  |  | Manufacturing, value added (% of GDP) | Stimulant | 2016-2020 |
|  |  | Net capital account (BoP, current US$) | Stimulant | 2016-2020 |
|  |  | Net financial account (BoP, current US$) | Stimulant | 2016-2020 |
|  |  | Net primary income (BoP, current US$) | Stimulant | 2016-2020 |
|  |  | Net secondary income (BoP, current US$) | Stimulant | 2016-2020 |
|  |  | Net trade in goods (BoP, current US$) | Stimulant | 2016-2020 |
|  |  | Net trade in goods and services (BoP, current US$) | Stimulant | 2016-2020 |
|  | **Urban development** | Population density (people per sq. km of land area) | Stimulant | 2016-2020 |
|  |  | Population in largest city | Stimulant | 2016-2020 |
|  |  | Population in the largest city (% of urban population) | Stimulant | 2016-2020 |
|  |  | Population in urban agglomerations of more than 1 million (% of total population) | Stimulant | 2016-2020 |
|  |  | Urban land area (sq. km) | Stimulant | 2010* |
|  |  | Urban population | Stimulant | 2016-2020 |
|  |  | Urban population (% of total population) | Stimulant | 2016-2020 |
|  |  | Urban population growth (annual %) | Stimulant | 2016-2020 |
| **SOCIAL SUSTAINABILITY** | **Science & Technology** | Charges for the use of intellectual property, payments (BoP, current US$) | Stimulant | 2016-2020 |
|  |  | Charges for the use of intellectual property, receipts (BoP, current US$) | Stimulant | 2016-2020 |
|  |  | High-technology exports (% of manufactured exports) | Stimulant | 2016-2020 |
|  |  | High-technology exports (current US$) | Stimulant | 2016-2020 |
|  |  | High-technology exports (current US$) | Stimulant | 2015-2019 |
|  |  | Patent applications, residents | Stimulant | 2015-2019 |
|  |  | Patent applications, nonresidents | Stimulant | 2015-2019 |
|  |  | Research and development expenditure (% of GDP) | Stimulant | 2014-2018 |
|  |  | Researchers in R&D (per million people) | Stimulant | 2014-2018 |
|  |  | Scientific and technical journal articles | Stimulant | 2014-2018 |
|  |  | Technicians in R&D (per million people) | Stimulant | 2014-2018 |
|  | **Social** | Children in employment, total (% of children ages 7-14 | Destimulant | 2011-2015 |
|  |  | Employment to population ratio, 15+, total (%) (modeled ILO estimate) | Stimulant | 2016-2020 |
|  |  | GDP per person employed (constant 2017 PPP $) | Stimulant | 2016-2020 |
|  |  | Labor force participation rate, female (% of female population ages 15+) (modeled ILO estimate) | Stimulant | 2015-2019 |
|  |  | Labor force participation rate, male (% of male population ages 15+) (modeled ILO estimate) | Stimulant | 2015-2019 |
|  |  | Labor force participation rate, total (% of total population ages 15+) (modeled ILO estimate) | Stimulant | 2016-2020 |
|  |  | Labor force with advanced education (% of total working-age population with advanced education) | Stimulant | 2015-2019 |
|  |  | Labor force, female (% of total labor force) | Stimulant | 2015-2019 |
|  |  | Labor force, total | Stimulant | 2016-2020 |
|  |  | Unemployment, total (% of total labor force) (modeled ILO estimate) | Destimulant | 2016-2020 |
|  |  | Wage and salaried workers, female (% of female employment) (modeled ILO estimate) | Stimulant | 2015-2019 |
|  |  | Wage and salaried workers, male (% of male employment) (modeled ILO estimate) | Stimulant | 2015-2019 |
|  |  | Prevalence of moderate or severe food insecurity in the population (%) | Destimulant | 2015-2019 |
|  | **Food Security** | Prevalence of severe food insecurity in the population (%) | Destimulant | 2015-2019 |
|  |  | Prevalence of undernourishment (% of population) | Destimulant | 2015-2019 |
|  |  | Use of insecticide-treated bed nets (% of under-5 population) | Destimulant | 2015-2019 |

* Indicators with data available for a specific year or arranged as data covering a long time period
